# Directed Induction of Functional Multi-ciliated Cells in Proximal Airway Epithelial Spheroids from Human Pluripotent Stem Cells

**DOI:** 10.1016/j.stemcr.2015.11.010

**Published:** 2015-12-24

**Authors:** Satoshi Konishi, Shimpei Gotoh, Kazuhiro Tateishi, Yuki Yamamoto, Yohei Korogi, Tadao Nagasaki, Hisako Matsumoto, Shigeo Muro, Toyohiro Hirai, Isao Ito, Sachiko Tsukita, Michiaki Mishima

**Affiliations:** 1 Department of Respiratory Medicine, Graduate School of Medicine, Kyoto University, Kyoto 606-8507, Japan; 2 Laboratory of Biological Science, Graduate School of Frontier Biosciences and Graduate School of Medicine, Osaka University, Suita 565-0871, Japan

## Abstract

Multi-ciliated airway cells (MCACs) play a role in mucociliary clearance of the lung. However, the efficient induction of functional MCACs from human pluripotent stem cells has not yet been reported. Using carboxypeptidase M (CPM) as a surface marker of NKX2-1^+^-ventralized anterior foregut endoderm cells (VAFECs), we report a three-dimensional differentiation protocol for generating proximal airway epithelial progenitor cell spheroids from CPM^+^ VAFECs. These spheroids could be induced to generate MCACs and other airway lineage cells without alveolar epithelial cells. Furthermore, the directed induction of MCACs and of pulmonary neuroendocrine lineage cells was promoted by adding DAPT, a Notch pathway inhibitor. The induced MCACs demonstrated motile cilia with a “9 + 2” microtubule arrangement and dynein arms capable of beating and generating flow for mucociliary transport. This method is expected to be useful for future studies on human airway disease modeling and regenerative medicine.

## Highlights

• CPM is a useful marker for generating human proximal airway epithelium
• Three-dimensional culture is useful for inducing human airway epithelium in vitro
• DAPT promotes the induction of multi-ciliated and pulmonary neuroendocrine cells
• Induced multi-ciliated airway cells have functionally motile cilia

## Graphical Abstract

**Figure:**
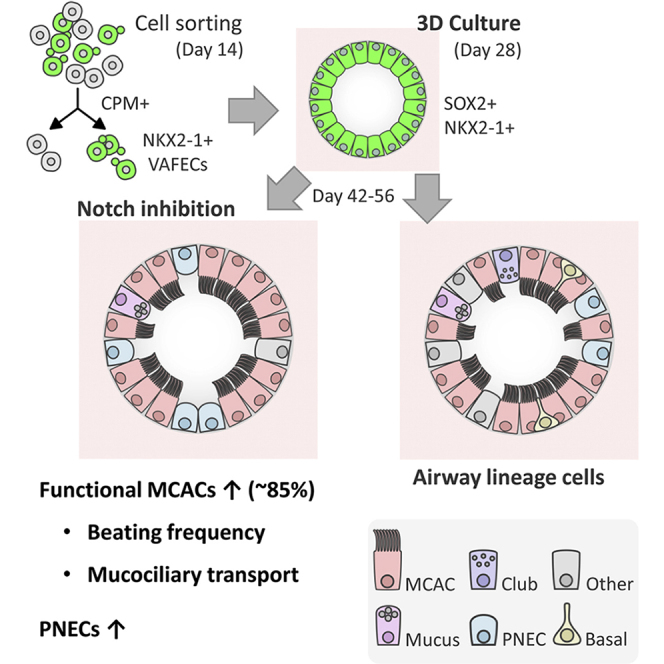

## Teaser

Multi-ciliated airway cells (MCACs) play a crucial role in mucociliary clearance. Gotoh, Konishi, and colleagues demonstrated that three-dimensional (3D) differentiation of CPM^+^ ventralized anterior foregut endoderm cells is useful for generating proximal airway epithelium, involving functional MCACs as well as pulmonary neuroendocrine cells (PNECs). The induction of MCACs and PNECs was promoted by adding DAPT, a Notch pathway inhibitor.

## Introduction

Proximal airway epithelial cells (PAECs) play a pivotal role in the host defense in the respiratory tract via mucociliary clearance organized by multi-ciliated airway cells (MCACs) and secretory cells. An abnormal function of MCACs is associated with various lung diseases such as primary ciliary dyskinesia (PCD) (Rossman et al., 1980) and cystic fibrosis (CF) (Zhang et al., 2009). It has been reported that PAECs could be generated from human pluripotent stem cells (hPSCs) involving human embryonic stem cells (hESCs) and induced pluripotent stem cells (hiPSCs) (Mou et al., 2012, Wong et al., 2012, Huang et al., 2014, Firth et al., 2014). The ciliary movement of hPSC-derived MCACs has not yet been reported, although that of murine embryonic stem cell-derived MCACs has been reported (Nishimura et al., 2006, Shojaie et al., 2015). In our previous study, we identified carboxypeptidase M (CPM) as a surface marker of NKX2-1^+^ “ventralized” anterior foregut endoderm cells (VAFECs) and demonstrated the potency of CPM^+^ VAFECs to differentiate into alveolar type II cells (Gotoh et al., 2014). We hypothesized that PAECs could also be induced from CPM^+^ VAFECs, as all lung epithelial lineage cells have been reported to be differentiated from NKX2-1^+^ VAFECs (Kimura et al., 1996). We herein report a method of directed differentiation of hPSCs into MCACs and pulmonary neuroendocrine cells (PNECs) and functional analyses of the ciliary movement of hPSC-derived MCACs.

## Results

### Generation of SOX2^+^NKX2-1^+^ PAEPC Spheroids from CPM^+^ VAFECs in Three-Dimensional Culture

**Figure 1:**
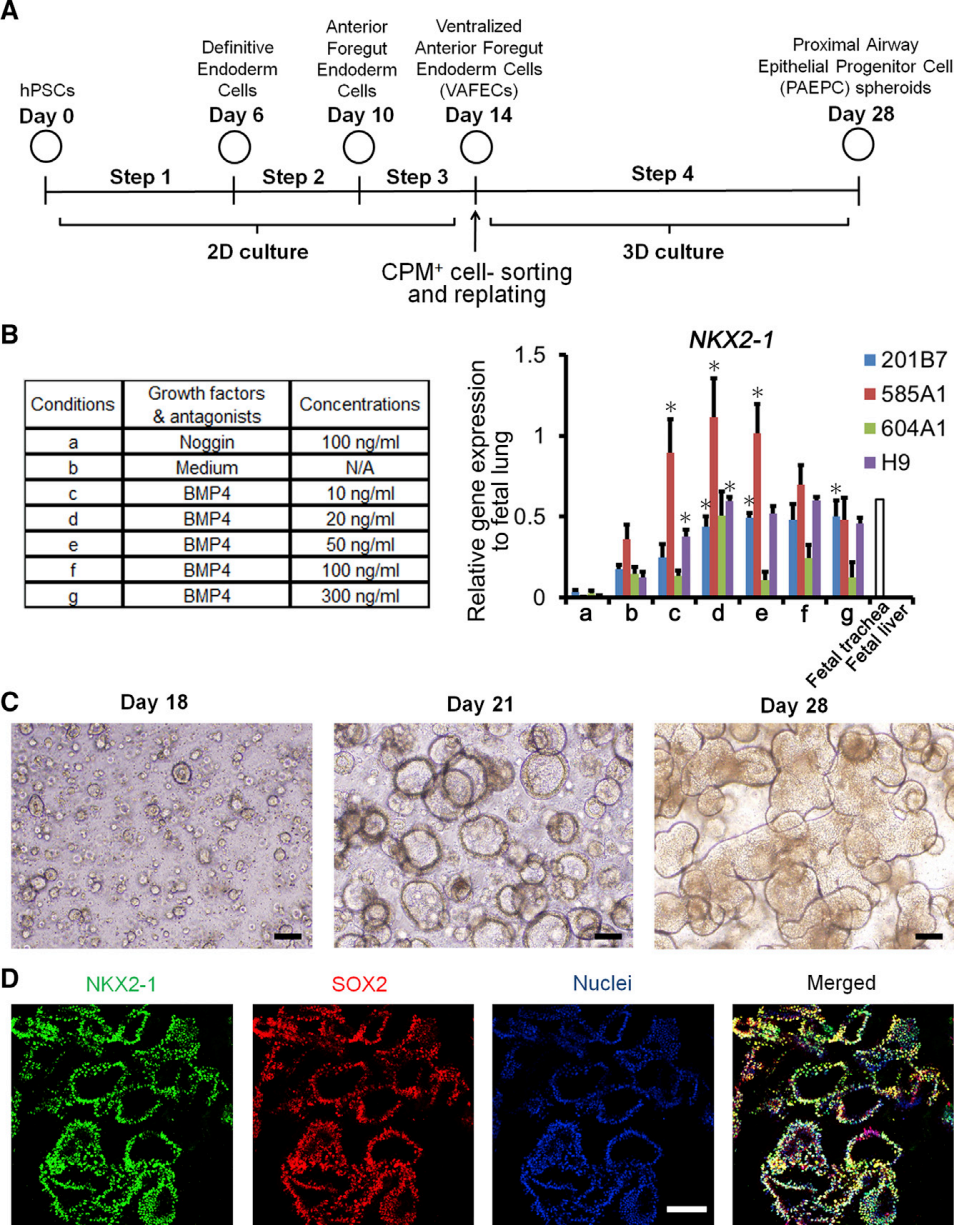
Generation of PAEPC Spheroids from CPM^+^ VAFECs in 3D Culture (A) Stepwise differentiation to PAEPC spheroids from hPSCs. (B) qRT-PCR of *NKX2-1* expression in each hPSC line on day 14, according to the dose of BMP4. The concentrations of BMP4 for each condition in Step 3 are shown in the columns. Each value was normalized to *β-ACTIN*. The gene expression level of the fetal lungs was set at 1. Error bars represent the mean ± SEM (n = 3 independent experiments). Each condition was compared with condition b for each hPSC line; ^∗^p < 0.05. N/A, not applicable. (C) CPM^+^ VAFEC-derived spheroids (201B7 hiPSCs) on days 18, 21, and 28. (D) CIF imaging shows 201B7 hiPSC-derived PAEPC spheroids coexpressing SOX2 and NKX2-1 on day 28. Scale bars, 100 μm. See also Figure S1 and Tables S1 and S2.

Because proximal airways develop as 3D branching structures in vivo, we adopted 3D differentiation from CPM^+^ VAFECs to proximal airway epithelial progenitor cells (PAEPCs) (Figure 1A). Undifferentiated hPSCs consisting of H9 hESCs (Thomson et al., 1998), 201B7 (Takahashi et al., 2007), 585A1, and 604A1 hiPSCs (Okita et al., 2013), were stepwise differentiated into NKX2-1^+^FOXA2^+^ VAFECs as previously reported (Gotoh et al., 2014), with the exception of the dose of BMP4 used in Step 3. We identified the minimal and sufficient dose of BMP4 to be 20 ng/ml for each hPSC line (Figure 1B). Interestingly, *NKX2-1* was downregulated in the presence of Noggin, which inactivates BMP signaling according to a quantitative RT-PCR (qRT-PCR) analysis. On day 14, CPM^+^ VAFECs were isolated and 3D culture was started in a similar manner as demonstrated in a tracheosphere assay using primary cells (Rock et al., 2009; Supplemental Experimental Procedures). In the hope of generating MCACs at the last step, the optimal medium conditions for proliferating spheroids and inducing *FOXJ1*, a representative marker of MCACs, were screened by combining FGF10, CHIR99021 (a WNT agonist), KGF, and DAPT (a γ-secretase inhibitor that blocks the Notch pathway), which have been considered to be important (Mou et al., 2012, Huang et al., 2014, Firth et al., 2014) (Figure S1A). The growth of the spheroids and *NKX2-1*, *SOX2*, and *FOXJ1* levels were compared on day 28 (Figures S1B and S1C), and the medium condition of 3 μM CHIR99021 and 100 ng/ml FGF10 was chosen. Under all conditions, *SOX9* was only slightly detected by qRT-PCR (Figure S1C). In Step 4, the spheroids grew larger and some of them began to fuse by day 28 (Figure 1C). Importantly, confocal immunofluorescence (CIF) imaging studies showed that nearly all the cells forming spheroids were SOX2^+^NKX2-1^+^ cells (Figure 1D), whereas SOX9 was not detected (data not shown), indicating that these cells were of PAEC lineage (Que et al., 2009).

### Derivation of PAECs from PAEPC Spheroids

**Figure 2:**
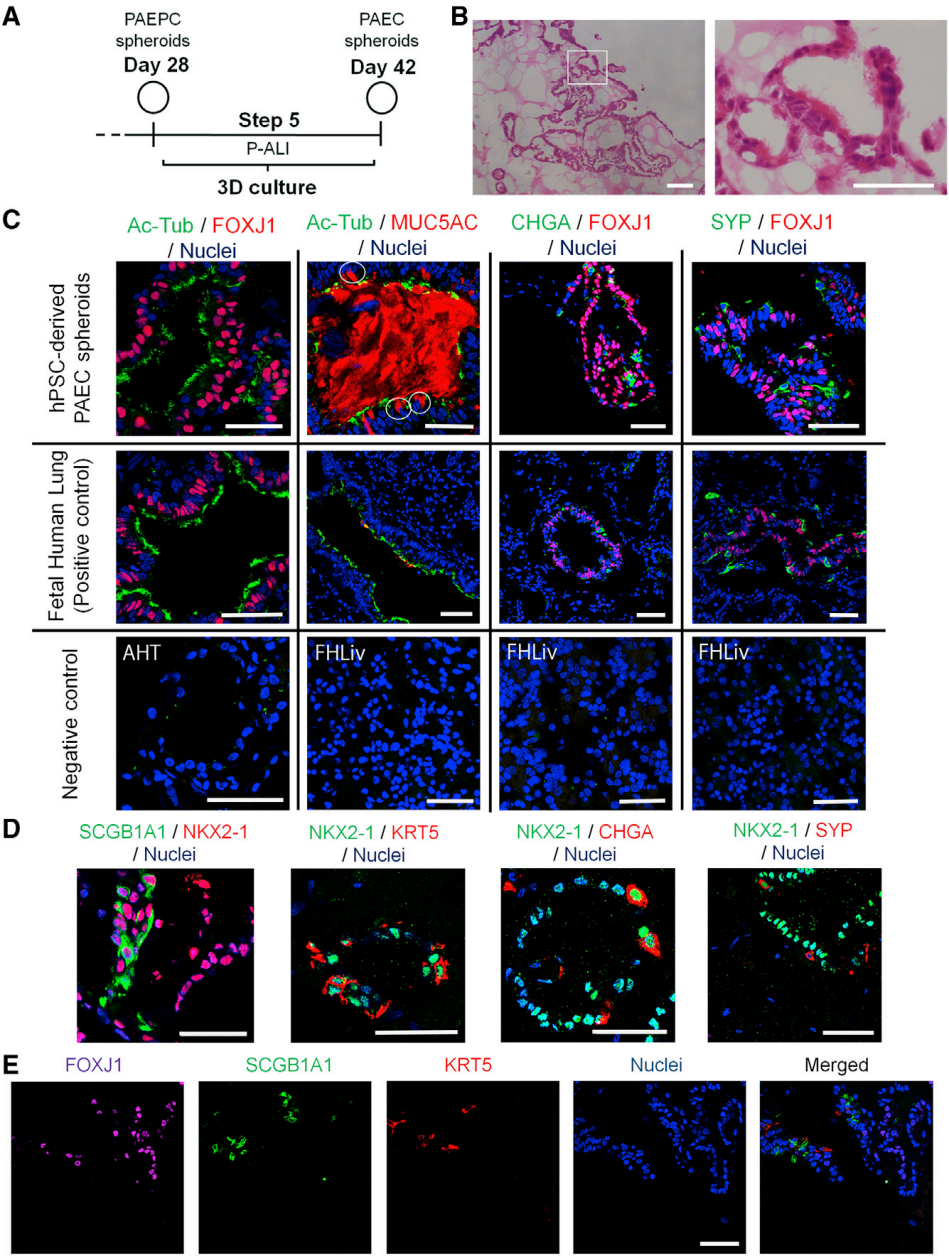
Derivation of PAECs from PAEPC Spheroids (A) A schematic illustration of the induction of PAECs from hPSC-derived PAEPC spheroids. (B) H&E staining of PAEC spheroids (201B7 hiPSCs) on day 42 shows spheroids (left) and clusters of MCACs in a magnified view (right). (C) Double immunostaining of PAEC spheroids (201B7 hiPSCs) on day 42. Fetal human lung was shown as a positive control. Adult human thyroid (AHT) was shown as a negative control for Ac-Tub and FOXJ1, whereas fetal human liver (FHLiv) was shown as a negative control for Ac-Tub, FOXJ1, MUC5AC, SYP, and CHGA. The white circle indicates MUC5AC^+^ cells. (D) Double immunostaining of induced PAEC spheroids (201B7 hiPSCs) on day 42. SCGB1A1, KRT5, CHGA, and SYP were detected in NKX2-1^+^ cells. (E) Triple immunostaining of PAEC spheroids (201B7 hiPSCs) on day 42. FOXJ1, SCGB1A1, and KRT5 were detected in the different cells. Scale bars, 50 μm. See also Figure S2 and Table S2.

At the end of Step 4, no MCACs were observed, which prompted us to hypothesize that there might be another step for inducing MCACs. Therefore, we switched the medium to Step 5 medium based on PneumaCult-ALI medium (P-ALI) (Stemcell Technologies), a medium for primary bronchial epithelial cells (Figure 2A). On day 42, clusters of MCACs were observed by H&E staining (Figure 2B). CIF imaging revealed acetylated tubulin (Ac-Tub)^+^FOXJ1^+^ cells and closely aligned Ac-Tub^+^ cells and MUC5AC^+^ cells, as observed in the fetal human lung (FHL), while secreted MUC5AC markedly accumulated in the closed lumen of the hPSC-derived spheroids (Figure 2C). A small number of SCGB1A1^+^cells (club cells), KRT5^+^cells (basal cells) and chromogranin A (CHGA)^+^ and synaptophysin (SYP)^+^cells (PNECs) were also found (Figure 2D). Nearly all the hPSC-derived PAECs expressed NKX2-1 (Figures 2D and S2A), consistent with the previous reports (Bilodeau et al., 2014) and CIF imaging of the FHL (Figure S2A). By triple immunostaining, each representative marker of MCACs, club cells and basal cells was expressed in the different cells (Figure 2E). FOXJ1^+^ cells did not overlap with the CHGA^+^ or SYP^+^ cells as in the FHL (Figure 2C). PGP9.5, another PNEC marker (Linnoila, 2006), was confirmed to be expressed in both CHGA^+^ and SYP^+^ cells (Figures S2B and S2C).

### DAPT Leads to the Efficient Induction of MCACs and Increases PNECs

**Figure 3:**
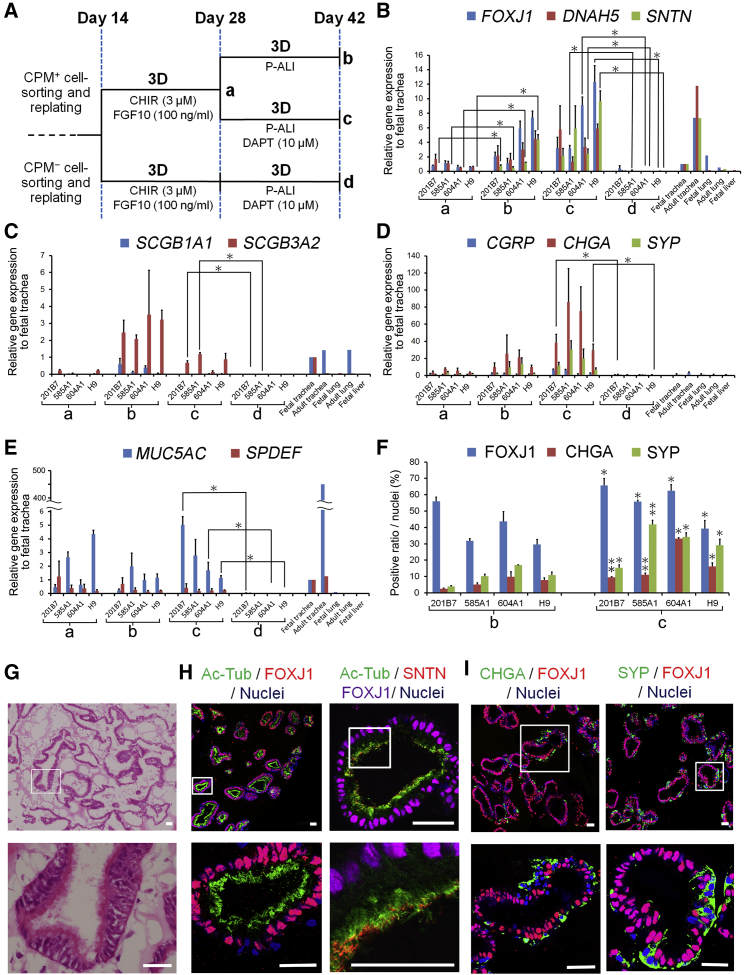
Directed Induction of MCACs and PNECs by Adding DAPT (A) A schematic illustration of each protocol for induction of PAECs, according to CPM-based sorting and the addition of DAPT. (B–E) qRT-PCR of representative PAEC markers: *FOXJ1*, *DNAH5*, and *SNTN* for MCACs (B), *SCGB1A1* and *SCGB3A2* for club cells (C), *CGRP*, *CHGA*, and *SYP* for PNECs (D), and *MUC5AC* and *SPDEF* for mucus-producing cells (E). Each value was normalized to *β-ACTIN*. The gene expression of the fetal trachea sample was set at 1. Error bars represent the mean ± SEM (n = 3 independent experiments; ^∗^p < 0.05). (F) The induction efficiency of MCACs and PNECs calculated by counting the number of FOXJ1^+^, CHGA^+^, and SYP^+^ cells (Supplemental Experimental Procedures). Error bars represent the mean ± SEM (n = 3 independent experiments). Protocol c was compared with protocol b for each hPSC line; ^∗^p < 0.05, ^∗∗^p < 0.01. (G) H&E staining of DAPT-induced 3D spheroids (201B7 hiPSCs) on day 56 (upper panel) showed consecutively aligned MCACs in a magnified view (lower panel). (H) Double and triple immunostaining of MCAC markers in DAPT-induced PAEC spheroids (201B7 hiPSCs) on day 56. Magnified views were shown in lower panels. (I) Double immunostaining of CHGA or SYP (PNEC markers) with FOXJ1 in DAPT-induced PAEC spheroids (201B7 hiPSCs) on day 56. None of the markers was expressed in FOXJ1^+^ cells. Scale bars, 25 μm. See also Figure S3 and Tables S1 and S2.

Because FOXJ1 is reportedly expressed before multi-ciliogenesis in vitro and in vivo (You et al., 2004, Rawlins et al., 2007), *SNTN*, which specifically marks MCACs (Kubo et al., 2008), was adopted to detect the suitable conditions for multi-ciliogenesis. *SNTN* was significantly increased on day 42 (Figure 3A, condition b), compared with day 28 (Figure 3A, condition a) in all hPSC lines (p < 0.05) (Figure 3B). In addition, each PAEC marker of MCACs (Figures 3B and S3B), club cells (Figures 3C and S3C), PNECs (Figures 3D and S3D), basal cells (Figure S3E), and mucus-producing cells (Figures 3E and S3F) increased after starting 3D culture (Figure S3A, condition a, b or both) compared with before 3D culture (days 6 and 14), while *AQP5* and *SFTPC* (alveolar type I and II cells, respectively) were almost negative (Figure S3G). *SFTPB* only slightly increased in accordance with an elevation of club cell markers (Figures 3C, S3C, and S3G, protocols a and b). *PAX6* (neuronal cells) and *PAX8* (thyroid cells) were also negative (data not shown). Next, the 3D protocol (Figure S3A, protocol a) was compared with the two-dimensional protocol (Figure S3A, protocol f) between days 14 and 28, resulting in an increase of some PAEC markers (Figures S3C, S3D, and S3F, protocols a and f). Because the cells spontaneously detached in 3D culture after day 28, three 3D protocols after the induction of VAFECs (Figure S3A, protocols b, c, and e) were compared with the four air-liquid interface (ALI) protocols (Figure S3A, protocols g–j), which involved two protocols modified from previous reports (Figure S3A, protocols i and j) (Wong et al., 2012, Firth et al., 2014; Supplemental Experimental Procedures).

DAPT was added to the media from days 28 to 42 (Figures 3A and S3A, protocol c and d) to increase hPSC-derived FOXJ1^+^ cells (Figure 3F). On day 42, the 3D protocols for CPM^+^ cells (Figures 3A and S3A, protocols b and c) appeared to induce higher gene expressions of MCAC and club cell markers than the 3D protocol for CPM^−^ cells (Figures 3A and S3A, protocol d) and ALI protocols (Figure S3A, protocols g–j), while the 3D protocol for CPM^−^ cells (Figures 3A and S3A protocol d) appeared to induce *KRT5* (a marker of both airway and esophageal basal cells), but not *NKX2-1* (Figure S3E). Importantly, *SNTN* increased only in the 3D protocols for CPM^+^ cells (Figures 3A and S3A, protocols b and c). Therefore, we concluded that the 3D protocols for CPM^+^ cells were beneficial for the induction of PAECs.

Next, we extended the culture period to day 56 (Figure S3A, protocol e), which increased *FOXJ1*, *DNAH5*, and *SNTN*. H&E staining and CIF imaging revealed that MCACs comprised a major part of the epithelia (Figures 3G and 3H). The rate of hPSC-derived FOXJ1^+^ cells was quantified on day 56 and compared with that on day 42, resulting in an increase in the ratio of FOXJ1^+^ cells to the total number of cells up to 85.65 ± 1.59% (p = 0.043), 85.82 ± 3.35% (p = 0.030), 72.7 ± 6.6% (p = 0.105), and 87.06 ± 0.43% (p = 0.011) in each hPSC line of 201B7, 585A1 and 604A1 hiPSCs, and H9 hESCs, respectively (n = 3 independent experiments). SNTN was localized at the tips of multiple cilia on day 56 (Figure 3H), which is consistent with the qRT-PCR results (Figures 3B and S3B). Moreover, hPSC-derived CHGA^+^ cells and SYP^+^ cells on day 42 (Figure 3A, protocols b and c) increased by adding DAPT (Figure 3F), consistent with the qRT-PCR results (Figures 3D and S3D, protocols b and c). Both the CHGA^+^ and SYP^+^ cells were localized to the aligning epithelium sparing FOXJ1^+^ cells (Figure 3I). In all the comparisons, the vehicle control (DMSO) was added to the media under the counterpart conditions in order to exclude the effects by DMSO solvent of DAPT.

### DAPT Suppresses the Notch Pathway in hPSC-Derived PNECs and Induces Functional Motile Cilia in hPSC-Derived MCACs

To elucidate the role of the Notch pathway in DAPT-induced differentiation of PNECs, NOTCH1 intracellular domain (N1ICD), HES1, and PGP9.5 were triply immunostained on day 42, and N1ICD^+^HES1^+^ cells were detected among the small number of non-PNECs (Figure S4A). By qRT-PCR, *DLL1* was significantly upregulated by DAPT in the H9 hESC line (p = 0.002), but not significantly in 201B7, 585A1, and 604A1 hiPSC lines (p = 0.114, 0.128, and 0.215, respectively). *HES1* was significantly suppressed by DAPT in the H9 hESC line (p = 0.013), but not significantly in 201B7, 585A1, and 604A1 hiPSC lines (p = 0.063, 0.225, and 0.44, respectively). *NOTCH1-3* on day 42 were unaffected, compatible with DAPT-mediated suppression of the Notch pathway (Figure S4B).

**Figure 4:**
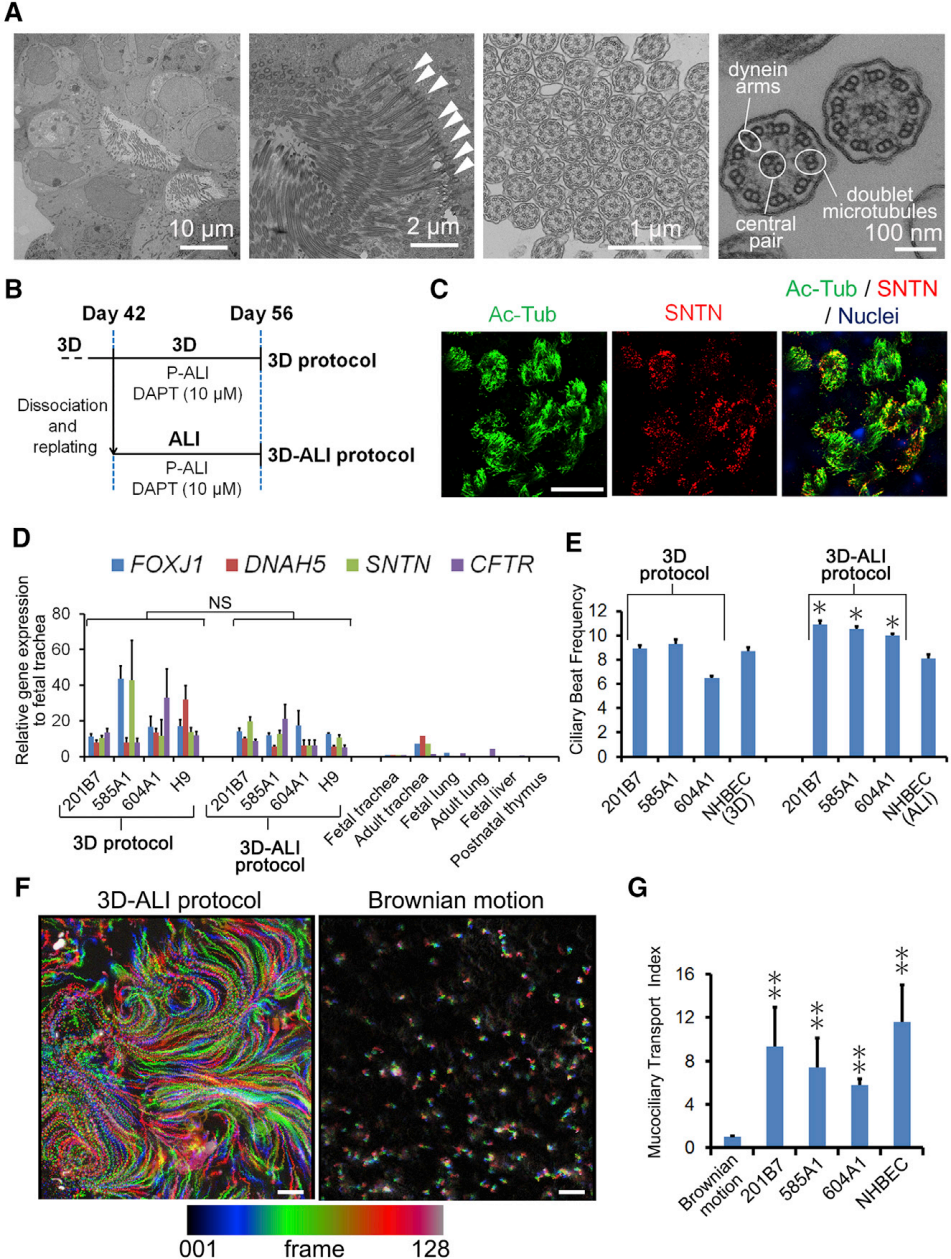
Characterization of Motile Cilia of hPSC-Derived MCACs (A) Transmission electron microscopy of DAPT-induced PAEC spheroids (201B7 hiPSCs) on day 56 (leftmost panel). Multiple cilia were originated from basal bodies (second left panel, arrowheads). A cross-sectional image of multiple cilia of induced MCACs (second right panel) was magnified to show the “9+2” structure with dynein arms (rightmost panel, circles). (B) A schematic illustration of the preparation of hPSC-derived MCACs for ciliary function tests. In the “3D protocol,” MCACs were differentiated until day 56 in spheroids. In the “3D-ALI protocol,” PAECs were dissociated from 3D Matrigel blocks on day 42, followed by replating and culturing under ALI condition until day 56. (C) Double immunostaining of Ac-Tub and SNTN in 201B7 hiPSC-derived MCACs cultured in the 3D-ALI protocol. (D) qRT-PCR of *FOXJ1*, *DNAH5*, *SNTN*, and *CFTR* expression in hPSC-derived MCACs cultured in the 3D and 3D-ALI protocols (n = 3 independent experiments). Each value was normalized to *β-ACTIN*. The gene expression of the fetal trachea sample was set at 1. NS, not significant. (E) The CBF of MCACs in spheroids and 3D-ALI condition in each hiPSC line in three independent experiments (n = 198, 135, and 314 cells in the spheroids and n = 174, 236, and 519 cells in the 3D-ALI condition derived from 201B7, 585A1, and 604A1 hiPSCs, respectively). NHBEC-derived MCACs were used as positive controls in each condition in three independent experiments (n = 123 and 86 in the 3D and the ALI condition, respectively). The CBF in the spheroids was compared with that in the 3D-ALI condition for each hiPSC line; ^∗^p < 0.05. (F) Stacked images of the fluorescent beads placed on 201B7 hiPSC-derived MCACs (left panel), and Brownian motion (right panel) acquired for 14.2 s. Color spectrum reflected time course. (G) The MTI of MCACs calculated from >100 trajectories of the fluorescent beads per sample in each hiPSC line in three independent experiments (n = 109, 142, 174, and 147 trajectories in 201B7, 585A1, and 604A1 hiPSCs and NHBECs, respectively). The MTIs in hiPSC-derived MCACs were compared with Brownian motion (n = 50) for each hiPSC line; ^∗∗^p < 0.01. Error bars in the qRT-PCR, CBF, and MTI analyses represent the mean ± SEM. Scale bars, 25 μm unless otherwise indicated. See also Figure S4; Movies S1 and S2; and Tables S1 and S2.

Next, the morphology of hPSC-derived MCACs was examined using electron microscopy, demonstrating multiple cilia originating from individual basal bodies on the apical surface of columnar epithelial cells (Figures 4A and S4C) and a “9+2” structure consisting of nine doublet and a central pair of singlet microtubules with dynein arms (Figure 4A, rightmost panel), which are specific features of motile cilia (Gibbons and Rowe, 1965).

On light microscopy, beating cilia were easily observed in the lumen of the spheroids and recorded by a high-speed camera (Movie S1, left panel). Metachronal wave-like beating of the cilia (Machemer, 1972) was observed in some MCACs (Figure S4D). In order to quantify the mucociliary flow over the MCACs, we established a protocol of passaging hPSC-derived MCACs in PAEC spheroids to ALI condition (Figure 4B, 3D-ALI protocol) due to the difficulty in measuring the flow rate inside the 3D spheroids. On day 56 of the 3D-ALI protocol, ciliary beating was observed on the apical side of MCACs (Movie S1, right panel). SNTN was localized at the tips of multiple cilia (Figure 4C), and CFTR was detected in the apical surface of MCACs (Figure S4E) and *FOXJ1*, *DNAH5*, *SNTN*, and *CFTR* levels appeared to be slightly, but not significantly, lower in the 3D-ALI protocol than in the 3D protocol (Figure 4D).

The ciliary beating frequency (CBF) was calculated by acquiring bright-field images of MCACs in the spheroids and the 3D-ALI condition based on the concepts previously described (Sisson et al., 2003) (Figure S4F; Supplemental Experimental Procedures). The CBF of each hiPSC line (201B7, 585A1, and 604A1)-derived MCACs showed 8.9 ± 0.27, 9.3 ± 0.34, and 6.5 ± 0.17 Hz in the spheroids on day 42 and 10.9 ± 0.31, 10.5 ± 0.26, and 10.0 ± 0.17 Hz in the 3D-ALI condition, respectively. A similar CBF was calculated for normal human bronchial epithelial cell (NHBEC)-derived MCACs in each condition (8.7 ± 0.30 Hz in the spheroids and 8.1 ± 0.33 Hz in the ALI condition) (Figure 4E). To measure mucociliary transport, the fluorescent beads placed on MCACs were traced (Movie S2; Figure 4F; Supplemental Experimental Procedures). The estimated flow velocity of the beads was approximately 7.4–10.1 μm/s in both hPSC- and NHBEC-derived MCACs. However, the values appeared to be affected by the lack of synchroneity of ciliary beating for generating a unidirectional flow (Movie S2; Figure 4F). Therefore, we analyzed the diffusion of the beads from their trajectories based on the concepts in a previous report (Qian et al., 1991). We defined the diffusion coefficient normalized to Brownian motion as the mucociliary transport index (MTI) (Supplemental Experimental Procedures). Then, the MTIs in the hPSC- and NHBEC-derived MCACs were calculated, and all the hPSC-derived MCACs showed slightly smaller MTIs compared with NHBEC-derived MCACs and significantly greater MTIs compared with Brownian motion (Figure 4G).

## Discussion

We established a method of 3D differentiation without feeder cells to generate hPSC-derived PAEC spheroids via isolated progenitor cells using CPM as a surface antigen, which is reportedly a biomarker of lung diseases, such as acute pneumonia and lung cancer (Dragović et al., 1995). It is noted that the inhibition of the Notch pathway induced not only MCACs but also PNECs from hPSCs, which is consistent with the studies of genetic murine models (Tsao et al., 2009, Morimoto et al., 2012). PNECs have been proposed to be the origin of small-cell lung cancer (Song et al., 2012), thus suggesting its future application in cancer studies.

The ciliary function analyses of hPSC-derived MCACs, as well as induction efficiency, are important aspects of the present study. Previously, the functional analyses of hPSC-derived PAECs mostly focused on CFTR (Wong et al., 2012, Firth et al., 2014), and not on ciliary movement. In addition, the ciliary function was not shown in hPSC-derived lung organoids due to immaturity (Dye et al., 2015). In the ciliary function tests, the CBF of hPSC-derived MCACs in spheroids appeared to be lower than that in the 3D-ALI protocol (Figure 4E) for at least two reasons. First, mucoid secretion was trapped in the closed lumen and its increased viscosity might reduce the CBF in the spheroids (Figure 2C and Movie S1, left). Second, we had to mince the spheroids and place cover slips on the samples during image acquisition, which may have reduced the CBF in the hPSC-derived spheroids, while we could directly observe the samples in the 3D-ALI protocol. The CBF of hPSC-derived MCACs in the 3D-ALI protocol was near the normal CBF of human MCACs, which range from 10 to 14 Hz (Rutland et al., 1982). Next, to quantify mucociliary transport, fluorescent beads were tracked as previously demonstrated in resected murine trachea (Kunimoto et al., 2012). Because synchronized ciliary beating for generating a unidirectional flow appeared to be incomplete in both hPSC- and NHBEC-derived MCACs (Movies S1 and S2; Figure 4F), as was reported for NHBEC-derived MCACs (Matsui et al., 1998), we further focused on the diffusion of the beads, demonstrating the potency of mucociliary clearance in hPSC-derived MCACs (Figures 4F and 4G). The difference between MCACs derived from hPSCs and NHBECs might be partly due to the difference in maturity. In addition, the ideal balance in the number of between MCACs and mucus-producing cells for mucociliary clearance remains to be elucidated. *MUC5AC* and *SPDEF* levels on day 56 were lower than on day 42 (Figure S3F), which might be due to differentiation (Chen et al., 2009) and/or apoptosis. In this respect, the regulation of MUC5AC^+^ cells by modulating factors, such as IL-13 (Atherton et al., 2003), remains to be a future subject. In conclusion, the findings of the present study are thus considered to pave the way for future applications toward modeling airway diseases, such as PCD and CF, or developing methods of airway reconstruction such as an artificial trachea.

## Experimental Procedures

### Imaging for the CBF and MTI

To measure the CBF, movies of hPSC- and NHBEC-derived MCACs were captured on a high-speed camera (FASTCAM MC2.1; Photron) connected to an upright microscope (Zeiss Axioplan; Carl Zeiss) with ×63 objectives. To measure MTI, the flow of fluorescent beads (Fluoresbrite, 0.5 μm; Polysciences) was recorded by an Orca-ER CCD camera (Hamamatsu) connected to an upright fluorescent microscope (BX51; Olympus) with a ×20 objective. See the Supplemental Experimental Procedures.

### Ethics

The use of H9 hESCs was approved by the Ministry of Education, Culture, Sports, Science, and Technology (MEXT) of Japan. For the use of human samples, human ethics approval was obtained from the Institutional Review Board and Ethics Committee of Kyoto University Graduate School and Faculty of Medicine.

### Statistical Analysis

At least three independent experiments were conducted in each study. The values are expressed as the means ± SEM. A two-tailed t test was performed to determine the statistical significance. p < 0.05 was considered to be significant.

## Author Contributions

S.G., S.K., K.T., and S.T. designed the study. S.K., K.T., S.G., Y.Y., Y.K., and T.N., performed the experiments. S.G., S.K., K.T., T.N., and S.T. analyzed the data. S.G. and S.K. wrote the manuscript through a fruitful discussion with and supervision by K.T., H.M., S.M., T.H., I.I., S.T., and M.M.

## Supplemental Information

Document S1. Figures S1–S4, Tables S1, S2, and Supplemental Experimental Procedures

Movie S1. Ciliary Beating of hiPSC-Derived MCACs, Related to Figure 4

Movie S2. Stacked Images of the Fluorescent Beads to Measure Mucociliary Transport, Related to Figure 4

Document S2. Article plus Supplemental Information
