# Supplementary material for: Directed Induction of Functional Multi-ciliated Cells in Proximal Airway Epithelial Spheroids from Human Pluripotent Stem Cells
Source: Stem Cell Reports. 2015 Dec 24;6(1):18–25. doi: 10.1016/j.stemcr.2015.11.010 (PMC4720023; doi:10.1016/j.stemcr.2015.11.010)
Supplement: Document S1. Figures S1–S4, Tables S1, S2, and Supplemental Experimental Procedures [file mmc1.pdf]

Stem Cell Reports, Volume *6*

Supplemental Information

Directed Induction of Functional Multi-ciliated Cells in Proximal Airway Epithelial Spheroids from Human Pluripotent Stem Cells

Satoshi Konishi, Shimpei Gotoh, Kazuhiro Tateishi, Yuki Yamamoto, Yohei Korogi, Tadao Nagasaki, Hisako Matsumoto, Shigeo Muro, Toyohiro Hirai, Isao Ito, Sachiko Tsukita, and Michiaki Mishima

Figure S1, related to Figure 1

A

Spheroid forming conditions in Step 4	Growth Factors / Concentration			
	CHIR99021	FGF10	KGF	DAPT
**(1)**	N/A	100 ng/ml	N/A	N/A
**(2)**	1 μM	100 ng/ml	N/A	N/A
**(3)**	3 μM	N/A	N/A	N/A
**(4)**	3 μM	10 ng/ml	N/A	N/A
**(5)**	3 μM	100 ng/ml	N/A	N/A
**(6)**	3 μM	10 ng/ml	10 ng/ml	N/A
**(7)**	3 μM	100 ng/ml	10 ng/ml	N/A
**(8)**	3 μM	100 ng/ml	N/A	10 μM
**(9)**	3 μM	100 ng/ml	N/A	50 μM

B

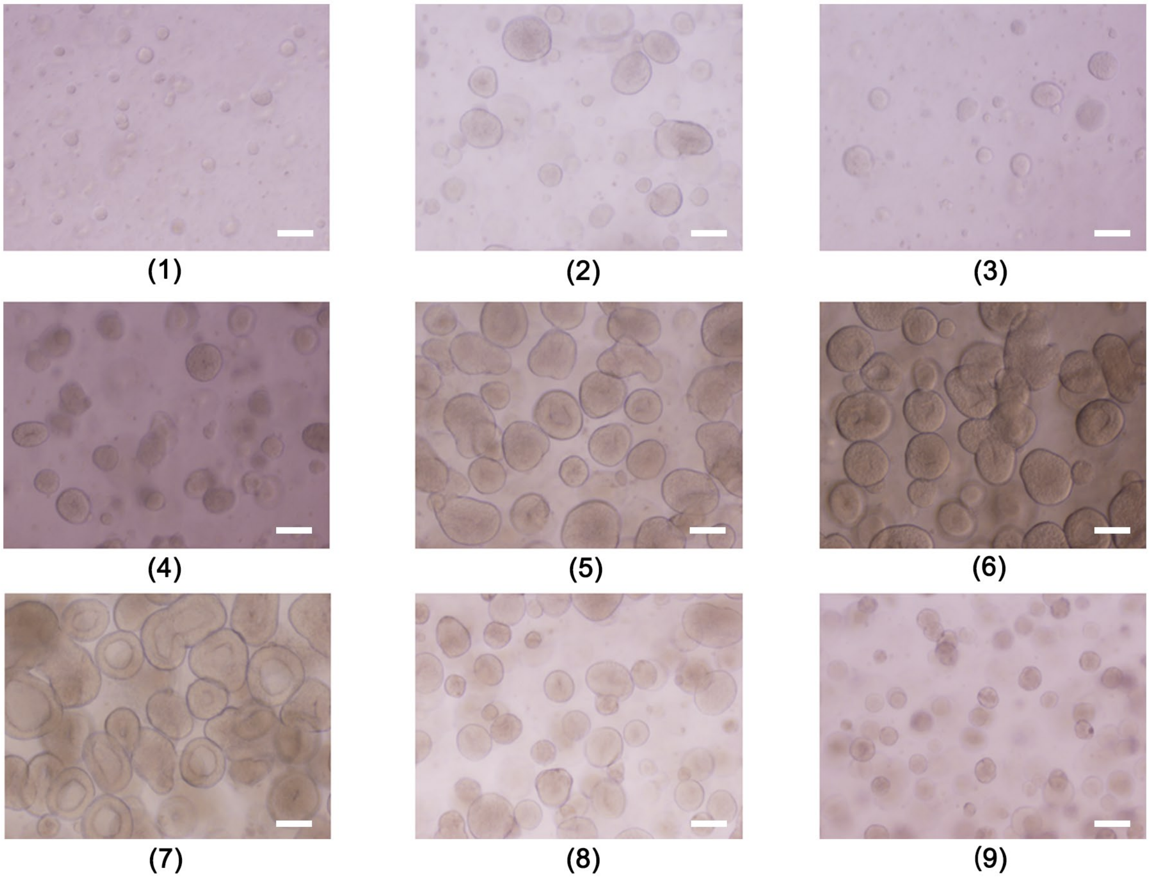

C

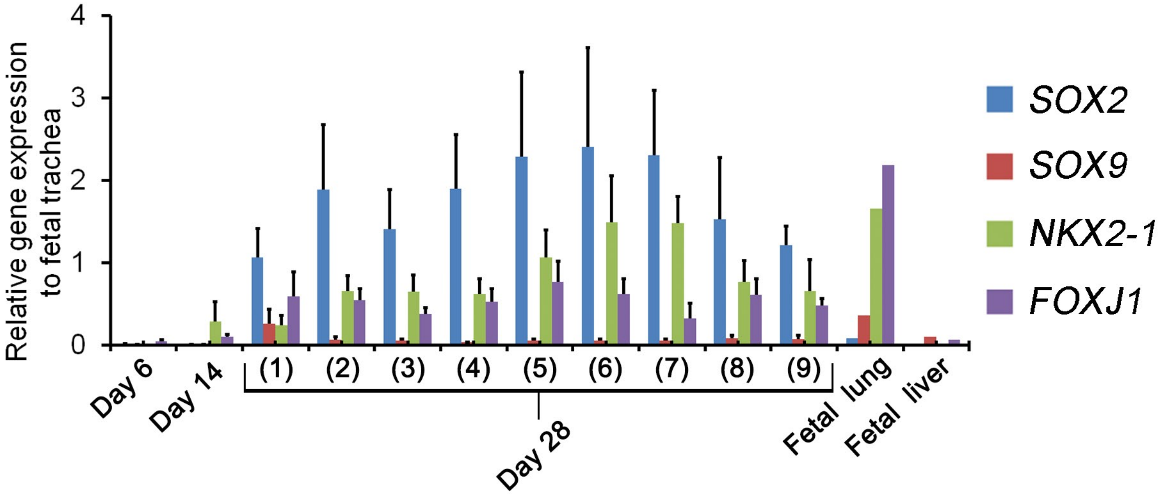

Figure S2, related to Figure 2

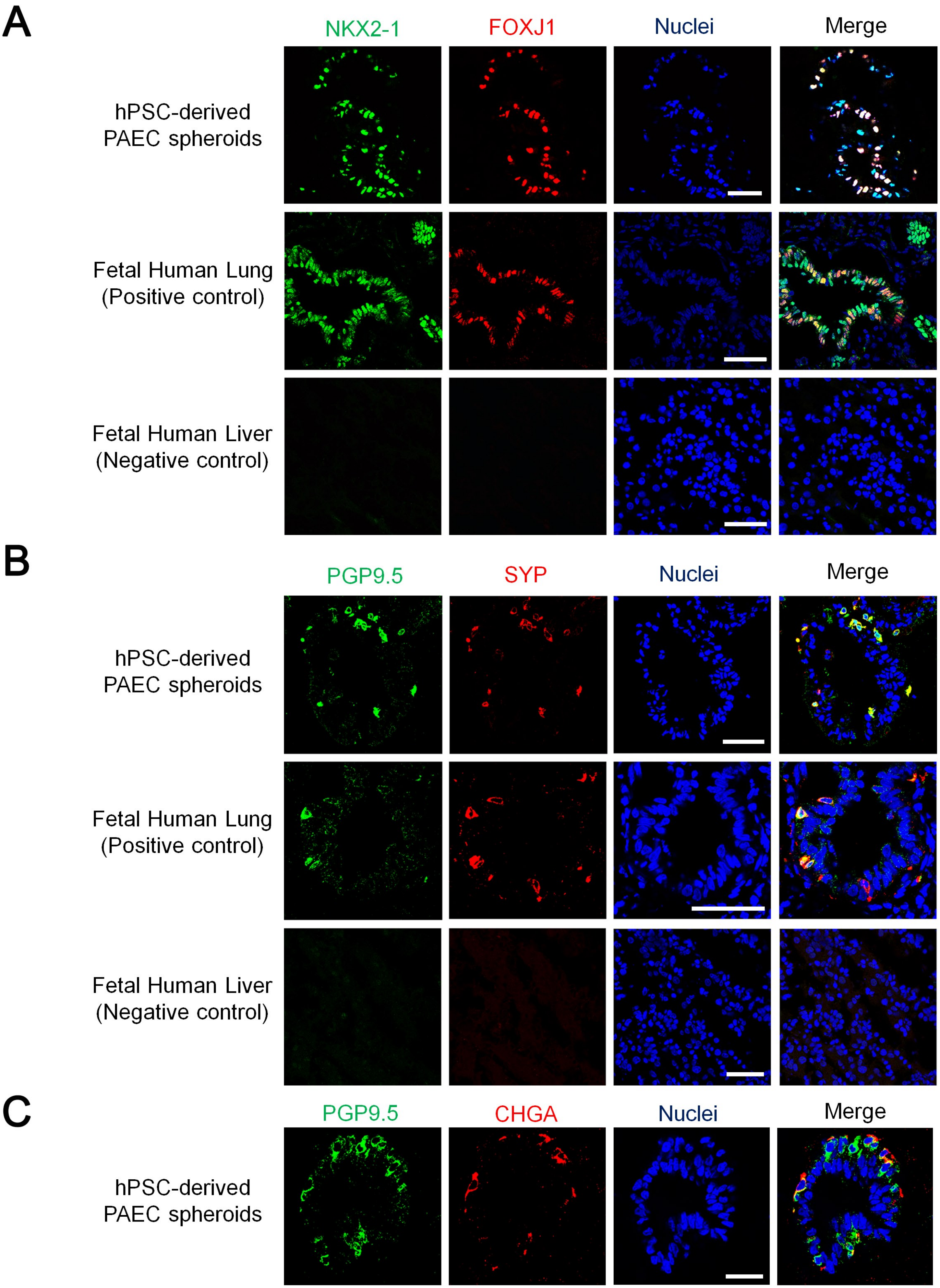

Figure S3, related to Figure 3

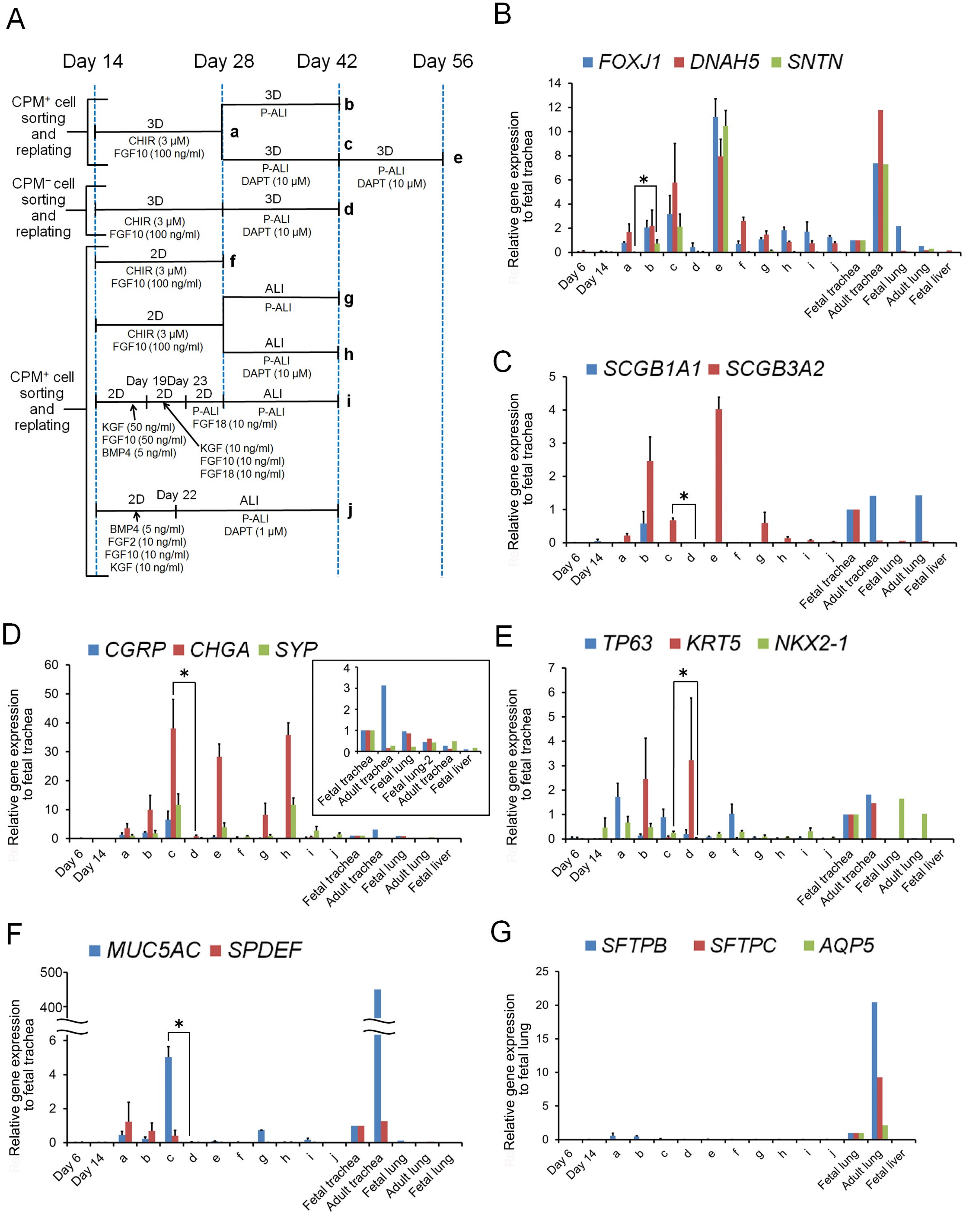

Figure S4, related to Figure 4

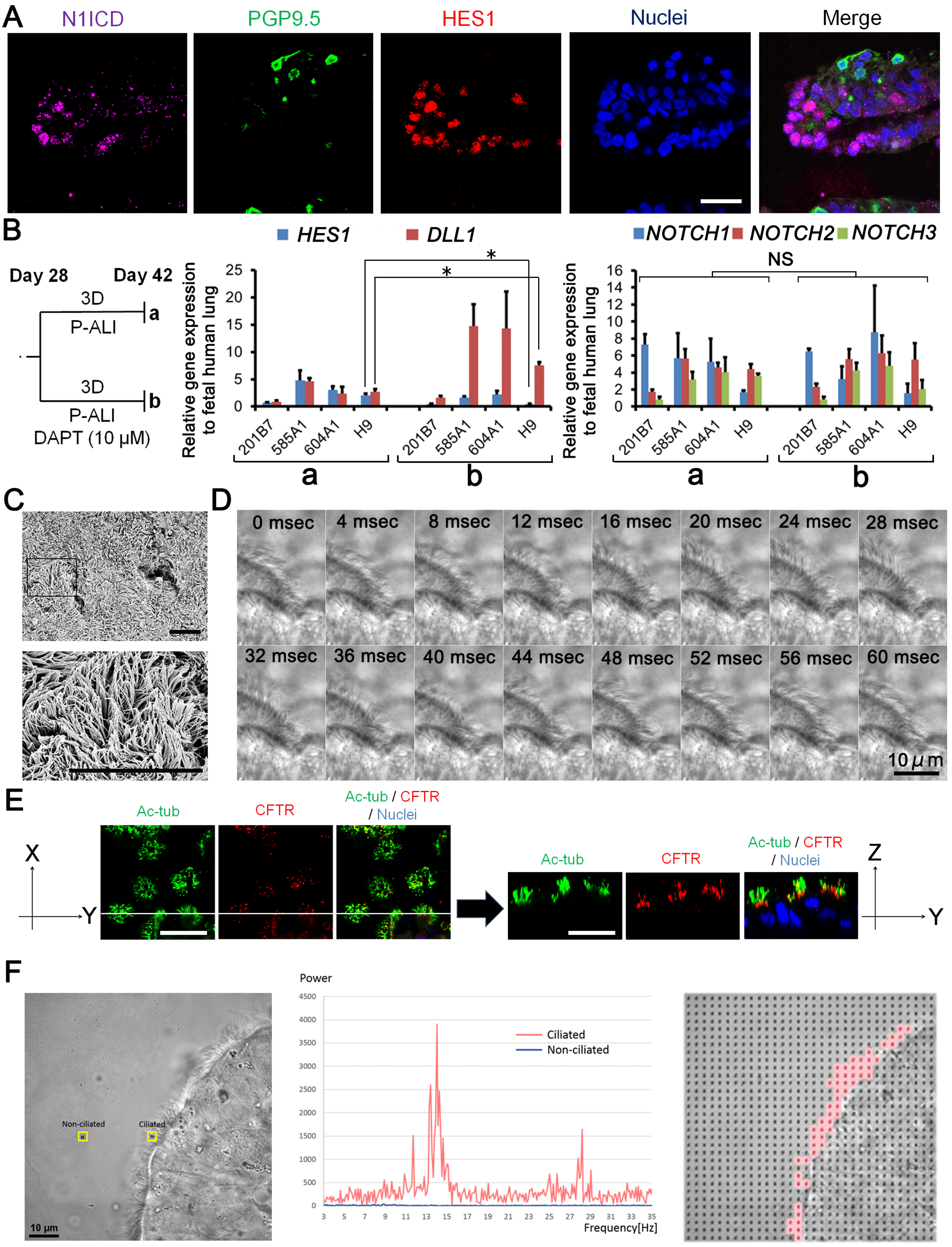

SUPPLEMENTAL FIGURE LEGENDS

Figure S1. Screening of medium conditions for forming PAEPC spheroids, related to Figure 1.

(A) Each medium condition evaluated in 3D culture in Step 4 from day 14 to day 28. (B) Representative images of the spheroids (201B7 hiPSCs) formed under each condition. Scale bars, 100 μm. (C) qRT-PCR of *SOX2, SOX9, NKX2-1* and *FOXJ1* in each condition at the end of Step 4 (day 28) (n=3 independent experiments) in 201B7 hiPSCs. Each value was normalized to *β-ACTIN*. The gene expression observed in the fetal trachea was set at 1. Error bars represent the mean ± SEM (n=3 independent experiments).

Figure S2. Characterization of hPSC-derived PAECs by immunostaining, related to Figure 2.

(A) Double immunostaining of NKX2-1 and FOXJ1 in PAEC spheroids (201B7 hiPSCs) on day 42 (top panels), fetal human lung as a positive control (middle panels), and fetal human liver as a negative control (bottom panels). Scale bars, 50 μm. (B) Double immunostaining of PGP9.5 and SYP in PAEC spheroids (585A1 hiPSCs) on day 42 (top panels), fetal human lung as a positive control (middle panels), and fetal human liver as a

negative control (bottom panels). PGP9.5 was positive in nearly all the SYP^+ cells. Scale bars, 50 μm. (C) Double immunostaining of PGP9.5 and CHGA in PAEC spheroids (585A1 hiPSCs) on day 42. Nearly all the $CHGA^+$ cells expressed PGP9.5, whereas some portion of $PGP9.5^+$ cells did not express CHGA.

Scale bars, 50 μm.

Figure S3. Comparison of gene expression of various lung lineage markers, related to Figure 3.

(A) A schematic illustration of various conditions for inducing PAECs post day 14. The same basal medium as in Steps 2 and 3 was used from days 14 to 28 of protocols a ~ i and from days 14 to 22 of protocol j. (B)~(G) qRT-PCR of each lineage cell marker in differentiated cells derived from 201B7 hiPSCs at the end of each condition (n=3 independent experiments): *FOXJ1*, *DNAH5* and *SNTN* (MCACs) (B), *SCGB1A1* and *SCGB3A2* (club cells) (C), *CGRP*, *CHGA* and *SYP* (PNECs) (D), *TP63* ,*KRT5* (basal cells), and *NKX2-1* (E), *MUC5AC* and *SPDEF* (mucus-producing cells) (F), and *SFTPB*, *SFTPC* and *AQP5* (alveolar epithelial cells) (G). Each value was normalized to *β-ACTIN*. The gene expression of the fetal human trachea sample was set at 1 in (B)~(F) and that of the fetal

human lung was set at 1 in (G). Error bars represent the mean ± SEM (n=3 independent experiments). In Figure S3D, the relative gene expressions of the positive controls (another fetal lung sample which was named as Fetal lung-2, was added) and the fetal human liver sample as a negative control were enlarged in the black square.

Figure S4. Characterization of hPSC-derived PNECs and MCACs, related to Figure 4.

(A) Triple immunostaining of PGP9.5, N1ICD and HES1 in DAPT-induced PAEC spheroids (585A1 hiPSCs) on day 42. Scale bar, 25 μm. (B) qRT-PCR of *HES1, DLL1*, *NOTCH1*, *NOTCH2* and *NOTCH3* of PAEC spheroids derived from each hPSC line on day 42. Each value was normalized to *β-ACTIN*. The gene expression level of the fetal lungs was set at 1. Error bars represent the mean ± SEM (n=3 independent experiments). NS means "not significant". *p<0.05. (C) Scanning electron microscopy of DAPT-induced PAEC spheroids (201B7 hiPSCs) on day 56 showed an apical surface of a spheroid covered by multiple cilia (left panel) with a magnified view (right panel). (D) Serially captured images of motile cilia of MCACs in a 3D spheroid (585A1 hiPSCs) on day 42 by a high-speed camera acquired at intervals of 4.0 ms. (E) Double immunostaining of Ac-Tub and CFTR in 201B7 hiPSC-derived PAECs cultured in the 3D-ALI protocol. Scale bar, 25

μm.

(F) A representative image of ROIs including a ciliated region or non-ciliated region in a spheroid (201B7 hiPSCs) on day 42 (left panel). Fast Fourier transform scores (FFT) obtained from the representative ROIs of the ciliated and non-ciliated regions in time-course (middle graph). ROIs involving the ciliated region were selected by FFT to calculate the CBF (pink area, right panel). Scale bar, 10 μm.

Movie S1. Ciliary beating of hiPSC-derived MCACs, related to Figure 4.

Beating cilia of MCACs in a spheroid (585A1 hiPSCs) on day 42 (left panel) and in a sheet cultured in the 3D-ALI protocol (604A1 hiPSCs) on day 56 (right panel) observed by using light microscopy and shot at 250 frames per second (fps) on a high-speed video camera.

Movie S2. Stacked images of the fluorescent beads to measure mucociliary transport, related to Figure 4.

Representative stacked images of the fluorescent beads placed on MCACs (201B7 hiPSCs) in the 3D-ALI protocol on day 56 (left panel) and Brownian motion of the beads as a negative control (right panel).

Table S1. Primers used in the present study.

Gene name		Primer Sequence	Size (bp)
β-ACTIN	F	CAATGTGGCCGAGGACTTTG	126
	R	CATTCTCCTTAGAGAGAAGTGG	
NKX2-1	F	AGGACACCATGAGGAACAGC	160
	R	GCCATGTTCTTGCTCACGTC	
SOX2	F	GCACATGAAGGAGCACCCGGATTA	86
	R	CGGGCAGCGTGTACTTATCCTTCTT	
SOX9	F	GAGGAAGTCGGTGAAGAACG	337
	R	ATCGAAGGTCTCGATGTTGG	
FOXJ1	F	CCTGTCGGCCATCTACAAGT	94
	R	AGACAGGTTGTGGCGGATT	
DNAH5	F	GCAATTGTGGCTTCCTGTTT	109
	R	GGAGACCTCCAACAGCAAAA	
SNTN	F	GCTGCAAACCCAATTTAGGA	84
	R	TGCTCATCAAGTTCAGAAAGGA	
SCGB1A1	F	CACCATGAAACTCGCTGTCAC	147
	R	AGTTCCATGGCAGCCTCATAAC	
SCGB3A2	F	CAAGTGGAACCACTGGCTTG	198
	R	CCAGAGGTAAAGGTGCCAAC	
P63	F	ACTGCCAAATTGCAAAGACA	184
	R	TGACTAGGAGGGGCAATCTG	
KRT5	F	GAGCTGAGAAACATGCAGGA	82
	R	TCTCAGCAGTGGTACGCTTG	
MUC5AC	F	CATCTGCCAGCTGATTCTGA	129
	R	AAGACGCAGCCCTCATAGAA	
SPDEF	F	AAGTGCTCAAGGACATCGAGA	94
	R	AGGAGCCACTTCTGCACATT	
CGRP	F	GGGGTGTGGTGAAGAACAAC	168
	R	CCATGGAGCCTTTCCTACAA	
CHGA	F	CGGATCCTTTCCATTCTGAG	105
	R	ACCGCTGTGTTTCTTCTGCT	
SYP	F	TTTGTGAAGGTGCTGCAATG	125
	R	ACCTCGATGCTGAGGTCACT	
SFTPB	F	GAGCCGATGACCTATGCCAAG	133
	R	AGCAGCTTCAAGGGGAGGA	
SFTPC	F	GCAAAGAGGTCCTGATGGAG	178
	R	TGTTTCTGGCTCATGTGGAG	
AQP5	F	CTGTCCATTGGCCTGTCTGTC	248
	R	GGCTCATACGTGCCTTTGATG	
PAX6	F	CGGAGTGAATCAGCTCGGTG	301
	R	CCGCTTATACTGGGCTATTTTGC	
PAX8	F	TCAACCTCCCTATGGACAGCTG	137
	R	GAGCCCATTGATGGAGTAGGTG	
CFTR	F	GATACAGACAGCGCCTGGAA	110
	R	TGAAGCCAGCTCTCTATCCCA	
HES1	F	ATGACAGTGAAGCACCTCCG	103
	R	ACTCGCTGAAGCCGGCTC	
DLL1	F	CTCCTTCAGTCTGCCCGAC	142
	R	TGTTGCGAGGTCATCAGGAG	
NOTCH1	F	GTCACCCACGAGTGTGCC	128
	R	CAGTTGTAGGTGTTCACGCC	
NOTCH2	F	ATTGCAGTGTCGAGATGGCT	141
	R	CGGTTCTTCTCACAGGGGTC	
NOTCH3	F	CAAATGGAGGTCGTTGCACC	103
	R	GAGTGACAGGGGTCCTCCA	

Table S2. Antibodies used in the present study

Primary Antibodies	Dilution rate	Manufacturer	Clone / Cat. No.
NKX2-1	1:500	Novus Biologocals	EP1584Y / NB100-80062
NKX2-1	1:500	Lab Vision	8G7G3/1 / MS-699-P
CPM	1:500	Leica microsystems	1C2 / NCL-CPMm
SOX2	1:500	EMD-Millipore	AB5603
SOX9	1:20	R&D systems	AF3075
FOXJ1	1:500	eBioscience	2A5/14-9965-82
Acetylated tubulin	1:4000	Sigma-Aldrich	T7451
SNTN	1:100	Atlas Antibodies	HPA043322
SCGB1A1	1:25	R&D systems	MAB4218/394324
KRT5	1:100	Lab Vision	EP1601Y / RM-2106-S0
MUC5AC	1:100	Thermo Scientific	45M1/MS-145-P
CHGA	1:100	Santa Cruz	sc-13090
SYP	1:100	Santa Cruz	sc-9116
CFTR	1:20	R&D systems	13-1/MAB1660
PGP9.5	1:50	abcam	13C4/ab8189
HES1	1:100	abcam	2D2/ab119776
Notch1 antibody-Cleaved-Val1744（N1ICD)	1:100	abcam	ab52301

Secondary Antibodies	Dilution rate	Manufacturer	Cat. No.
Donkey anti-mouse IgG(H+L) (Alexa488)	1:500	Life Technoloies	A21202
Goat anti-mouse IgG2a (Alexa488)	1:500	Life Technoloies	A21131
Goat anti-mouse IgG2b (Alexa488)	1:500	Life Technoloies	A21141
Goat anti-mouse IgG1 (Cy3)	1:500	Jackson ImmunoResearch	115-165-205
Goat anti-mouse IgG (H+L) (Alexa647)	1:500	Life Technoloies	A21236
Goat anti-mouse IgG1 (Alexa647)	1:500	Life Technoloies	A21240
Rat anti-mouse IgG1 (microbeads)	1:5	Miltenyi Biotec	130-047-101
Goat anti-rabbit IgG (H+L) (Alexa488)	1:500	Life Technoloies	A11008
Donkey anti-rabbit IgG (H+L) (Alexa488)	1:500	Life Technoloies	A21206
Donkey anti-rabbit IgG (H+L) (Cy3)	1:500	Jackson ImmunoResearch	711-165-152
Donkey anti-rabbit IgG (H+L) (Alexa647)	1:500	Jackson ImmunoResearch	711-605-152
Donkey anti-rat IgG (H+L) (Alexa488)	1:500	Life Technoloies	A21208

SUPPLEMENTAL EXPERIMENTAL PROCEDURES

Culture of hPSCs

H9 hESCs and 201B7, 585A1 and 604A1 hiPSCs were generally cultured and maintained on mitomycin C-treated STO feeder cells in Primate ES medium (ReproCELL) as described previously (Gotoh et al., 2014).

For the 201B7 and 585A1 hiPSC lines, the cells were alternatively maintained on feeder-free Geltrex (Life Technologies)-coated plates in Essential 8 medium (Life Technologies) supplemented with 50 U/ml of penicillin/streptomycin (Life Technologies) prior to the differentiation studies within 10 passages. The cells cultured in the feeder-free system were passaged with a split ratio of 1:3 or 1:4 after being washed with PBS, incubated in 0.5mM EDTA/PBS for 5 minutes at 37 °C and suspended in pre-warmed Essential 8 medium.

2D differentiation of hPSCs into ventralized anterior foregut endoderm cells (VAFECs)

For the differentiation of hPSCs maintained on mitomycin C-treated STO feeder cells, 70% confluent hPSCs were dissociated and seeded as described previously (Gotoh et

al., 2014).

For the differentiation of feeder free hiPSCs, 80% confluent hiPSCs were incubated in 10 μM of Y-27632 (LC Laboratories) for 1 hour prior to dissociation. The cells were subsequently rinsed carefully with PBS and incubated in Accutase (Innovative Cell Technologies) for 20 minutes at 37°C. The detached hPSCs were then dissociated into single cells via pipetting, and seeded on Geltrex-coated plates at a density of 1.375 $x10^5$ cells/cm^2 in Step 1 medium containing RPMI1640 medium (Nacalai Tesque), 1x B27 supplement (Life Technologies, #17504-044), 50 U/ml of penicillin/streptomycin, 100 ng/ml of human activin A (R&D systems) and 1 μM of CHIR99021 (Axon Medchem), supplemented with 10 μM of Y-27632 (day 0-1) and 0.25 mM (day 1) and 0.125 mM (day 2-6) of sodium butyrate (Kajiwara et al., 2012). From Step 2 to Step 3, the basal medium consisted of DMEM/F12 plus GlutaMAX (Life Technologies), 1x B27 supplement, 50 U/ml of penicillin/streptomycin, 0.05 mg/ml of L-ascorbic acid (Wako) and 0.4 mM of monothioglycerol (Wako), as minimally modified from our previous report (Gotoh et al., 2014). On day 6, the medium was changed to Step 2 medium, containing the basal medium with 100 ng/ml of human recombinant Noggin (HumanZyme) and 10 μM of SB-431542 (Stem RD) (Green et al., 2011). On day 10, the medium was changed to Step 3 medium,

containing the basal medium with 20 ng/ml of human recombinant BMP4 (HumanZyme) and the combination of optimal doses of all-trans retinoic acid (ATRA) (Sigma-Aldrich) and CHIR99021; 0.5 μM and 3.5 μM for H9 hESCs, 0.05 μM/2.5 μM for 201B7 hiPSCs, 0.5 μM/3.5 μM for 585A1 hiPSCs and 1.0 μM/2.5 μM for 604A1 hiPSCs.

Isolation of CPM^+ and CPM^- cells from VAFECs

The induced VAFECs were dissociated with Accutase and resuspended in DMEM/F12 containing 2% FBS. The cells were filtered through a 40 μm cell strainer (BD Falcon) to remove cell clumps.

The dissociated cells were washed and resuspended in 1% BSA/PBS, followed by staining with mouse anti-human CPM antibodies (Leica Microsystems) at 4°C for 15 minutes. After rinsing twice with 0.5% BSA/PBS containing 2 mM EDTA, the cells were stained at 4°C for 15 minutes with anti-mouse IgG1 microbeads (Miltenyi Biotec) for CPM^+ cell-sorting or with Alexa 647-conjugated anti-mouse IgG antibody (Life Technologies) for CPM^- cell-sorting. We avoided to sort CPM^- cells by magnetic activated cell sorting (MACS), because a portion of the CPM^+ cells were involved in the CPM^- cell fraction as reported previously (Gotoh et al., 2014).

After washing, the cells were separated using a magnetic stainless column (Miltenyi Biotec) twice or FACSAria II flowcytometer (BD Biosciences). A total of 10 μM of Y-27632 was maintained for all processes until the CPM^+ cells were sorted.

Differentiation of CPM^+ VAFECs into proximal airway epithelial cell (PAEC) spheroids

A total of $4.0x10^5$ cells/cm^2 of CPM^+ cells isolated from the induced VAFECs on day 14 were resuspended in 112 μl of Step 4 medium supplemented with 10 μM of Y-27632 and mixed with an equal volume of growth factor-reduced Matrigel (Coning). In 3D differentiation protocols (Figures 3 and S3, protocols a ~ e), a total volume of 224 μl was carefully pipetted into a 12-well cell culture insert (Corning, #353180) and 1 ml of Step 4 medium was added to the lower chamber and changed every other day for 14 days. On day 28, the medium of the lower chamber was changed to Step 5 medium and replaced every other day until day 42 or day 56.

Step 4 medium consisted of the same basal medium as Steps 2 and 3, and contained 3.0 μM of CHIR99021, 100 ng/ml of FGF10, and 10 μM of Y-27632.

Step 5 medium consisted of PneumaCult-ALI Maintenance medium (STEMCELL

Technologies) supplemented with 10 μM of Y-27632. PneumaCult-ALI Maintenance medium was prepared by adding the attached supplement solution, 4 μg/ml of heparin (Nacalai Tesque) and 1 μM of hydrocortisone (Sigma-Aldrich) to the ready-made basal medium, according to the manufacturer's instructions. 10 μM of Y-27632 was kept to avoid dissociation-induced cell death throughout the induction process from day 14 to the end (Watanabe et al., 2007). For comparison, two ALI protocols (Figure S3A, protocols i and j) were modified from previous reports (Wong et al., 2012; Firth et al., 2012). In one ALI protocol (Figure S3A, protocol i), the medium included FGF18 from day 19 to day 28 and the cells were cultured in ALI condition (Wong et al., 2012). In the other ALI protocol (Figure S3A, protocol j), the cells were cultured in medium containing DAPT as an inhibitor of Notch signaling pathway from day 22 to day 42 in ALI conditions (Firth et al., 2012).

Measurement of the ciliary beat frequency and mucociliary transport

To analyze ciliary beat frequency (CBF) and mucociliary transport, hiPSC-derived multi-ciliated airway cells (MCACs) which were differentiated in 3D Matrigel blocks were dissociated on day 42 and replated on a Geltrex-coated 12-well cell culture insert in a total

volume of 0.5 ml at a density of 7.5 $x10^5$ cells/cm^2 and 1 ml of Step 5 medium plus 10 μM of DAPT was added to the lower chamber and replaced every other day for 14 days before analyses. By reducing the medium to 0.125 ml in the upper chamber, ALI condition was started on day 43. Each sample was placed on the surface of a slide glass and dipped in 100 μl of Step 5 medium.

In the 3D-ALI protocol, the CBF of MCACs in a sheet was examined using an upright microscope (Zeiss Axioplan; Carl Zeiss) with an x63 water immersion objective. In the 3D protocol, the CBF of MCACs in the spheroids was examined by mincing the 3D Matrigel blocks into small pieces on day 42, putting cover slips on the samples and observing ciliary movement using a Zeiss Axioplan microscope with a x63 oil immersion objective. Normal human bronchial epithelial cell (NHBEC) (Lonza) -derived MCACs that were induced in ALI culture or 3D Matrigel blocks in Step 5 medium plus 10 μM of DAPT for 14-28 days were used as positive controls.

For measuring the CBF, 2,048 frames of bright-field images of the beating cilia were acquired at 250 frames per second (fps) on a high-speed video camera (FASTCAM MC2.1; Photron) with the Photron FASTCAM Viewer Ver. 3.0 software program. Then the CBF was calculated using a fast Fourier transform (FFT) based analysis. First, each frame

was subdivided into 1,024 small regions of interests (ROIs) (2.5 x 2.5 μm square) in using the Image J software program (National Institutes of Health). Then, the average brightness was calculated for each ROI and frame, followed by FFT using Scilab software programs (Scilab Enterprises). To minimize the effect of noise, we selected ROIs that included beating cilia by thresholding the CBF within the range of 3 and 35 Hz. A representative image of the selected ROIs is shown in Figure S4F.

To analyze mucociliary transport, the flow of fluorescent beads (a 1000-fold dilution of Fluoresbrite, 0.5 μm; Polysciences) that reflected mucociliary transport was recorded at 9 fps and a total of 128 frames per take were acquired by an Orca-ER CCD camera (Hamamatsu) connected to an upright fluorescent microscope (BX51; Olympus) with x20 objective. Fluorescent beads placed on the ALI-induced MCACs were traced with MTrackJ (Image J plug-in). After confirming the estimated flow velocity of the beads to be approximately 7.4-10.1 μm/s in both hiPSC- and NHBEC-derived MCACs, we evaluated the degree of diffusion of the beads to avoid an inaccurate conclusion affected by velocity generated by fluctuating movements of the beads without orientation. Then, the mean square displacement (MSD) was calculated from the trajectories in each samples and applied to the Einstein-Smoluchowski equation;

$$< r^2 (t) > = 2dDt$$

$< r^2 (t) >$ was regarded as the MSD. Dimension (d) was set as 2, because each trajectory was obtained by two-dimensional image acquisition in the present study. Time (t) was set as 14.2 sec required to take 128 frames at 9 fps. The resulting diffusion coefficient (D) was defined as the mucociliary transport index in the present study. Experiments were performed using hiPSC (201B7, 585A1 and 604A1 hiPSCs)- and NHBEC-derived MCACs for three times independently per cell line. Brownian motion was set as a negative control in analyzing mucociliary transport (Codling et al., 2008). In each experiment, more than 100 cells and 100 trajectories in each hiPSC line, were analyzed for calculating the CBF and mucociliary transport index, respectively. The samples were carried using a portable CO_2 incubator (Corefront) and observed immediately after taking out of the incubators to keep a condition of 5% CO_2 at 37°C.

Quantitative RT-PCR

Total RNA was isolated using the PureLink RNA Mini Kit (Life Technologies). cDNA was synthesized from 80 ng of total RNA using the SuperScript III First-Strand Synthesis System (Life Technologies) and amplified using Power SYBR PCR Master Mix

with the ABI7300 Real-Time PCR System (Life Technologies), as described previously (Gotoh et al., 2014). The PCR reactions were performed in duplicate for each sample. The expression level of each gene was calibrated to that of *β-ACTIN* and compared to the expression level of each gene in the fetal human lung samples (17, 18, and 22 weeks of gestation, Agilent Technologies, #540177, Lot.0006055802), or fetal trachea samples (29 weeks of gestation, Agilent Technologies, #R1244160-10, Lot.B402231). All primer sets are shown in Table S1.

CIF imaging

The fetal human tissue cryosections and cells cultured under 2D condition were fixed with 4% paraformaldehyde /PBS (Nacalai Tesque) for 15 minutes at RT. For immunostaining of Matrigel-embedded 3D spheroids, the samples were fixed with 4% paraformaldehyde for 15 minutes at RT, then after washing with PBS three times, left in 30% sucrose overnight at 4°C. The samples were subsequently embedded in the OCT compound (Sakura Finetek) and sectioned at 10 μm slices. After washing three times with PBS, the samples were permeabilized in 0.2% Triton X-100 /PBS for 15 minutes at RT and blocked with 5% normal donkey serum (Millipore)/1% BSA/PBS for 30 minutes at RT.

Finally, the cells were immunostained with the primary antibodies for 30 minutes at RT and with the secondary antibodies for 30 minutes at RT, as described previously (Gotoh et al., 2014). All primary and secondary antibodies are listed in Table S2. The nuclei were counterstained with Hoechst-33342. All immunofluorescence images were acquired using a TCS SP8 confocal microscope (Leica Microsystems) in order to analyze co-localization of cell lineage markers precisely. The quantification of efficiency was performed by scoring the number of FOXJ1$^+$ cells, CHGA$^+$ cells or SYP$^+$ cells relative to the total number of nuclei using the MetaMorph 7.7 image analysis software program (Molecular Devices) in an average of five randomly selected fields at 20x magnification (n = 3 independent experiments).

Electron microscopy

Matrigel-embedded epithelial spheroids were incubated in fixative containing 2.5% glutaraldehyde, 4% paraformaldehyde and 0.1M phosphate buffer (pH 7.4) at RT for two hours. For transmission electron microscopy, because the Matrigel-embedded spheroids were slurry and did not easily form pellets, they were re-embedded in the agarose gel. After being washed three times in 0.1 M phosphate buffer (pH 7.4), the samples were

incubated in 1 % osmium tetroxide (Nacalai Tesque) for two hours. Then, the samples were washed in ascending concentrations of ethanol and embedded in epoxy-resin and DMP-30 (Nacalai Tesque). Thin sections were doubly stained with uranyl acetate and lead citrate and examined under a Hitachi H-7650 transmission electron microscope. For scanning electron microscopy, the samples were post fixed in 1 % osmium tetroxide (Nacalai Tesque) for two hours. The samples were then dehydrated, dried, and coated with a thin layer of platinum palladium. Finally, the specimens were examined with a Hitachi S-4700 scanning electron microscope.

Human fetal tissues and total RNA samples

The following human specimens were obtained from DV Biologics (Canada), Agilent Technologies (United States), and BioChain (United States): total RNA of the fetal lungs (17, 18 and 22 weeks of gestation, Agilent Technologies, #540177, Lot.0006055802), another sample of the fetal lung for confirming the gene expression of PNEC markers (36 weeks of gestation, BioChain, #R1244152-50, Lot.B210105), adult lung (40 years of age, Agilent Technologies, #540019, Lot.0006118369), fetal trachea (29 weeks of gestation, Agilent Technologies, #R1244160-10, Lot.B402231), adult trachea (29 years of age,

BioChain, # R1234160-50, Lot.B803066), fetal liver (20 weeks of gestation, BioChain, # R1244149-50, Lot.A601605), and postnatal thymus (6, 6, and 10 months of age, Life Technologies, #AM6000, Lot.1102082), and frozen tissue of the fetal lung (18.5 weeks of gestation, DV Biologics, PP001-FS, Lot.102508RH), fetal liver (28 weeks of gestation, BioChain, T1244149, Lot.B511025) and adult thyroid (25 years of age, BioChain, #T1234265-RT1, Lot.A709031).

SUPPLEMENTAL REFERENCES

Codling EA, Plank MJ, Benhamou S. (2008). Random walk models in biology. J R Soc Interface. *5*, 813-834.

Green MD, Chen A, Nostro MC, d'Souza SL, Schaniel C, Lemischka IR, Gouon-Evans V, Keller G, Snoeck HW. (2011). Generation of anterior foregut endoderm from human embryonic and induced pluripotent stem cells. Nat. Biotechnol. *29*, 267–272.

Kajiwara M, Aoi T, Okita K, Takahashi R, Inoue H, Takayama N, Endo H, Eto K, Toguchida J, Uemoto S, Yamanaka S. (2012). Donor-dependent variations in hepatic differentiation from human-induced pluripotent stem cells. Proc. Natl. Acad. Sci. USA.*109,* 12538-12543.

Watanabe K, Ueno M, Kamiya D, Nishiyama A, Matsumura M, Wataya T, Takahashi JB, Nishikawa S, Nishikawa S, Muguruma K, Sasai Y. (2007). A ROCK inhibitor permits survival of dissociated human embryonic stem cells. Nat. Biotechnol. *25*, 681-686.
