# Supplementary figures and images for: Directed Induction of Functional Multi-ciliated Cells in Proximal Airway Epithelial Spheroids from Human Pluripotent Stem Cells
Source: Stem Cell Reports. 2015 Dec 24;6(1):18–25. doi: 10.1016/j.stemcr.2015.11.010 (PMC4720023; doi:10.1016/j.stemcr.2015.11.010)

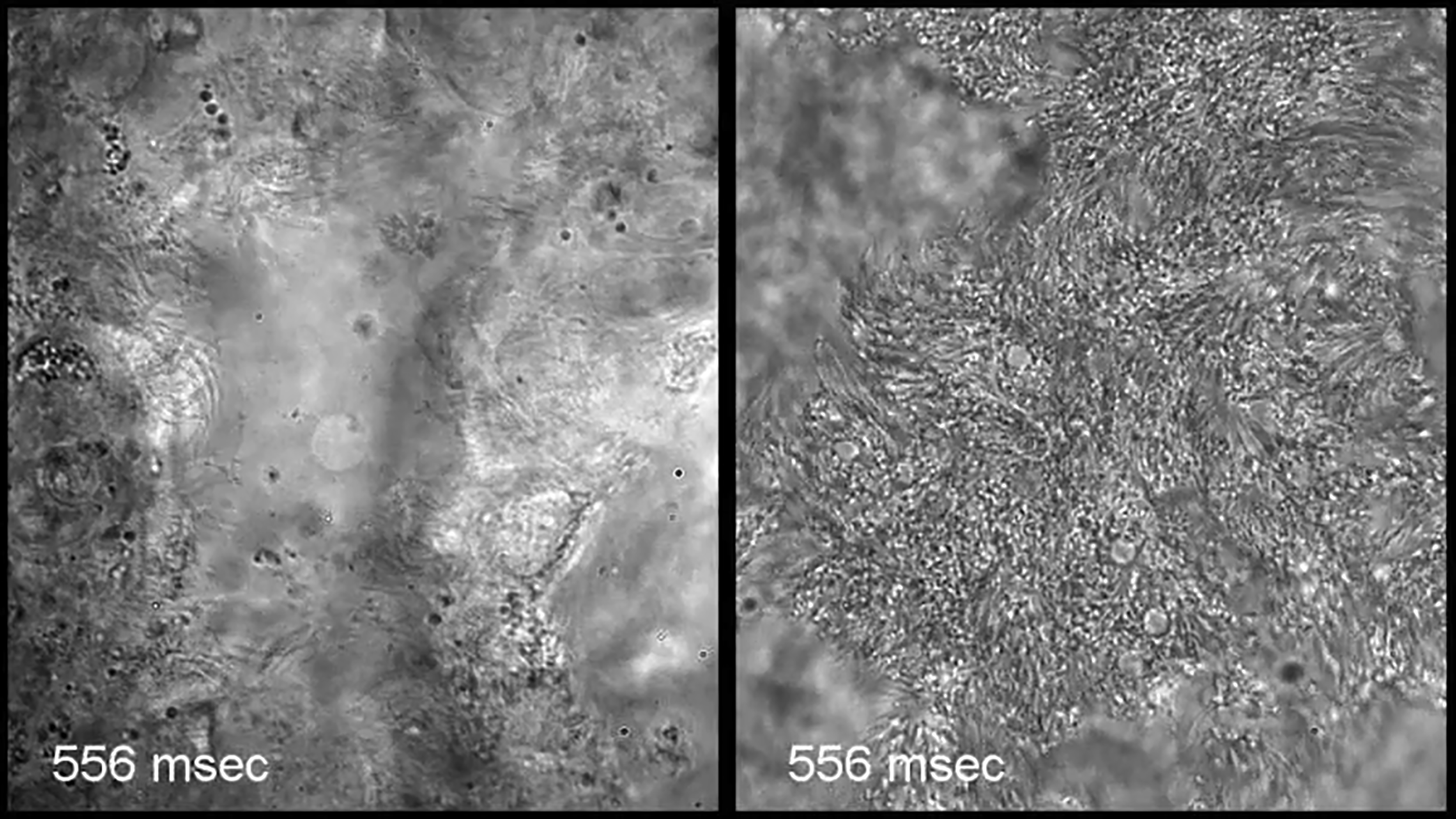

Supplement: Movie S1. Ciliary Beating of hiPSC-Derived MCACs, Related to Figure 4 [file mmc2.jpg]

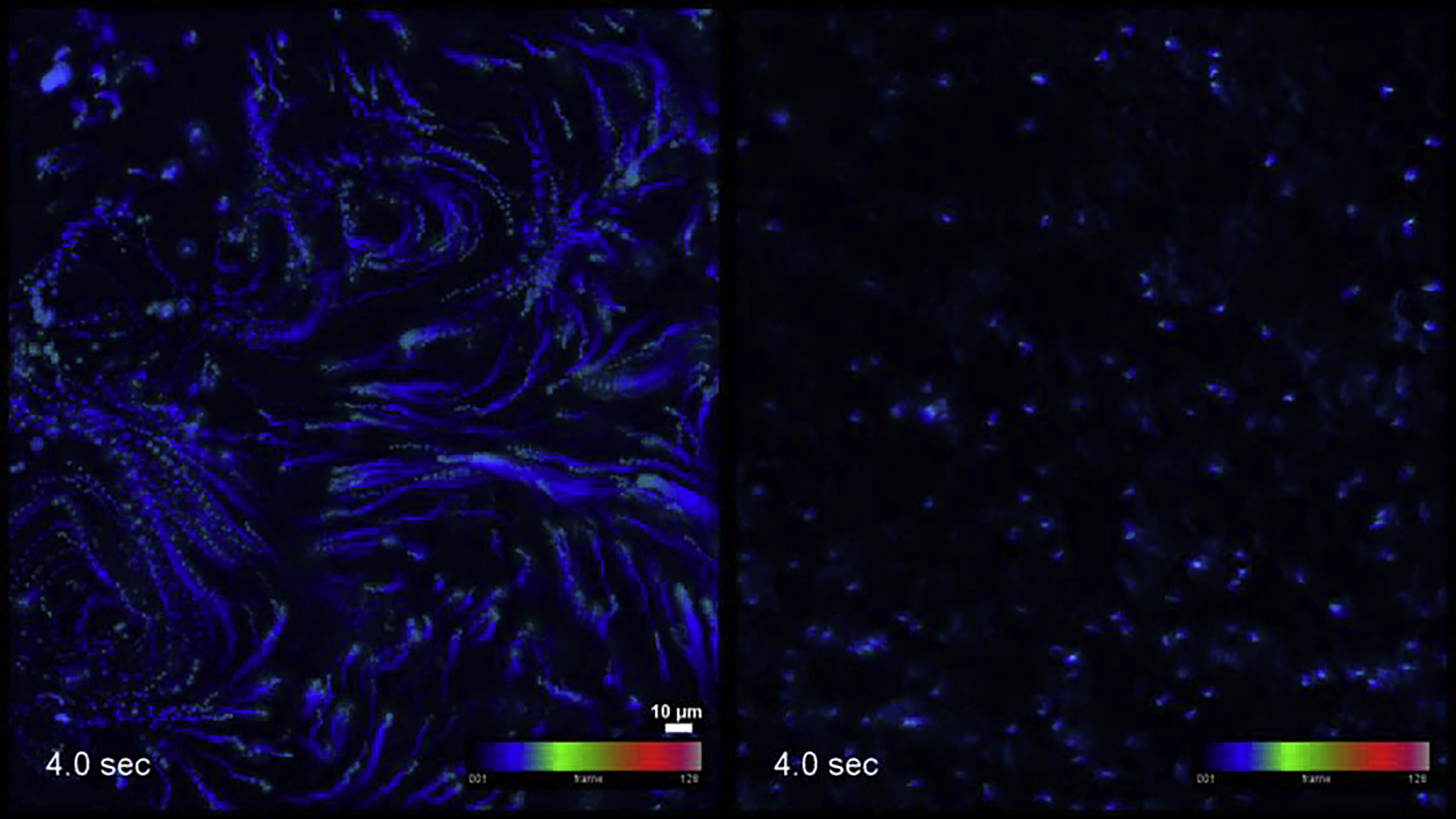

Supplement: Movie S2. Stacked Images of the Fluorescent Beads to Measure Mucociliary Transport, Related to Figure 4 [file mmc3.jpg]
