# Supplementary material for: Directed Induction of Functional Multi-ciliated Cells in Proximal Airway Epithelial Spheroids from Human Pluripotent Stem Cells
Source: Stem Cell Reports. 2015 Dec 24;6(1):18–25. doi: 10.1016/j.stemcr.2015.11.010 (PMC4720023; doi:10.1016/j.stemcr.2015.11.010)
Supplement: Document S2. Article plus Supplemental Information [file mmc4.pdf]

Stem Cell Reports
Report

ISSCR
OPEN ACCESS

Directed Induction of Functional Multi-ciliated Cells in Proximal Airway Epithelial Spheroids from Human Pluripotent Stem Cells

Satoshi Konishi,[1] Shimpei Gotoh,[1,*] Kazuhiro Tateishi,[2] Yuki Yamamoto,[1] Yohei Korogi,[1] Tadao Nagasaki,[1] Hisako Matsumoto,[1] Shigeo Muro,[1] Toyohiro Hirai,[1] Isao Ito,[1] Sachiko Tsukita,[2] and Michiaki Mishima[1]
[1]Department of Respiratory Medicine, Graduate School of Medicine, Kyoto University, Kyoto 606-8507, Japan
[2]Laboratory of Biological Science, Graduate School of Frontier Biosciences and Graduate School of Medicine, Osaka University, Suita 565-0871, Japan
*Correspondence: a0009650@kuhp.kyoto-u.ac.jp
http://dx.doi.org/10.1016/j.stemcr.2015.11.010
This is an open access article under the CC BY license (http://creativecommons.org/licenses/by/4.0/).

SUMMARY

Multi-ciliated airway cells (MCACs) play a role in mucociliary clearance of the lung. However, the efficient induction of functional MCACs from human pluripotent stem cells has not yet been reported. Using carboxypeptidase M (CPM) as a surface marker of NKX2-1^{+}-ventralized anterior foregut endoderm cells (VAFECs), we report a three-dimensional differentiation protocol for generating proximal airway epithelial progenitor cell spheroids from CPM^{+} VAFECs. These spheroids could be induced to generate MCACs and other airway lineage cells without alveolar epithelial cells. Furthermore, the directed induction of MCACs and of pulmonary neuroendocrine lineage cells was promoted by adding DAPT, a Notch pathway inhibitor. The induced MCACs demonstrated motile cilia with a “9 + 2” microtubule arrangement and dynein arms capable of beating and generating flow for mucociliary transport. This method is expected to be useful for future studies on human airway disease modeling and regenerative medicine.

INTRODUCTION

Proximal airway epithelial cells (PAECs) play a pivotal role in the host defense in the respiratory tract via mucociliary clearance organized by multi-ciliated airway cells (MCACs) and secretory cells. An abnormal function of MCACs is associated with various lung diseases such as primary ciliary dyskinesia (PCD) (Rossman et al., 1980) and cystic fibrosis (CF) (Zhang et al., 2009). It has been reported that PAECs could be generated from human pluripotent stem cells (hPSCs) involving human embryonic stem cells (hESCs) and induced pluripotent stem cells (hiPSCs) (Mou et al., 2012; Wong et al., 2012; Huang et al., 2014; Firth et al., 2014). The ciliary movement of hPSC-derived MCACs has not yet been reported, although that of murine embryonic stem cell-derived MCACs has been reported (Nishimura et al., 2006; Shojaie et al., 2015). In our previous study, we identified carboxypeptidase M (CPM) as a surface marker of NKX2-1^{+} “ventralized” anterior foregut endoderm cells (VAFECs) and demonstrated the potency of CPM^{+} VAFECs to differentiate into alveolar type II cells (Gotoh et al., 2014). We hypothesized that PAECs could also be induced from CPM^{+} VAFECs, as all lung epithelial lineage cells have been reported to be differentiated from NKX2-1^{+} VAFECs (Kimura et al., 1996). We herein report a method of directed differentiation of hPSCs into MCACs and pulmonary neuroendocrine cells (PNECs) and functional analyses of the ciliary movement of hPSC-derived MCACs.

RESULTS

Generation of SOX2^{+}NKX2-1^{+} PAEPC Spheroids from CPM^{+} VAFECs in Three-Dimensional Culture

Because proximal airways develop as 3D branching structures in vivo, we adopted 3D differentiation from CPM^{+} VAFECs to proximal airway epithelial progenitor cells (PAEPCs) (Figure 1A). Undifferentiated hPSCs consisting of H9 hESCs (Thomson et al., 1998), 201B7 (Takahashi et al., 2007), 585A1, and 604A1 hiPSCs (Okita et al., 2013), were stepwise differentiated into NKX2-1^{+}FOXA2^{+} VAFECs as previously reported (Gotoh et al., 2014), with the exception of the dose of BMP4 used in Step 3. We identified the minimal and sufficient dose of BMP4 to be 20 ng/ml for each hPSC line (Figure 1B). Interestingly, *NKX2-1* was downregulated in the presence of Noggin, which inactivates BMP signaling according to a quantitative RT-PCR (qRT-PCR) analysis. On day 14, CPM^{+} VAFECs were isolated and 3D culture was started in a similar manner as demonstrated in a tracheosphere assay using primary cells (Rock et al., 2009; Supplemental Experimental Procedures). In the hope of generating MCACs at the last step, the optimal medium conditions for proliferating spheroids and inducing *FOXJ1*, a representative marker of MCACs, were screened by combining FGF10, CHIR99021 (a WNT agonist), KGF, and DAPT (a γ-secretase inhibitor that blocks the Notch pathway), which have been considered to be important (Mou et al., 2012; Huang et al., 2014; Firth et al., 2014) (Figure S1A). The growth of the spheroids and *NKX2-1*, *SOX2*, and *FOXJ1* levels were compared on day

CrossMark

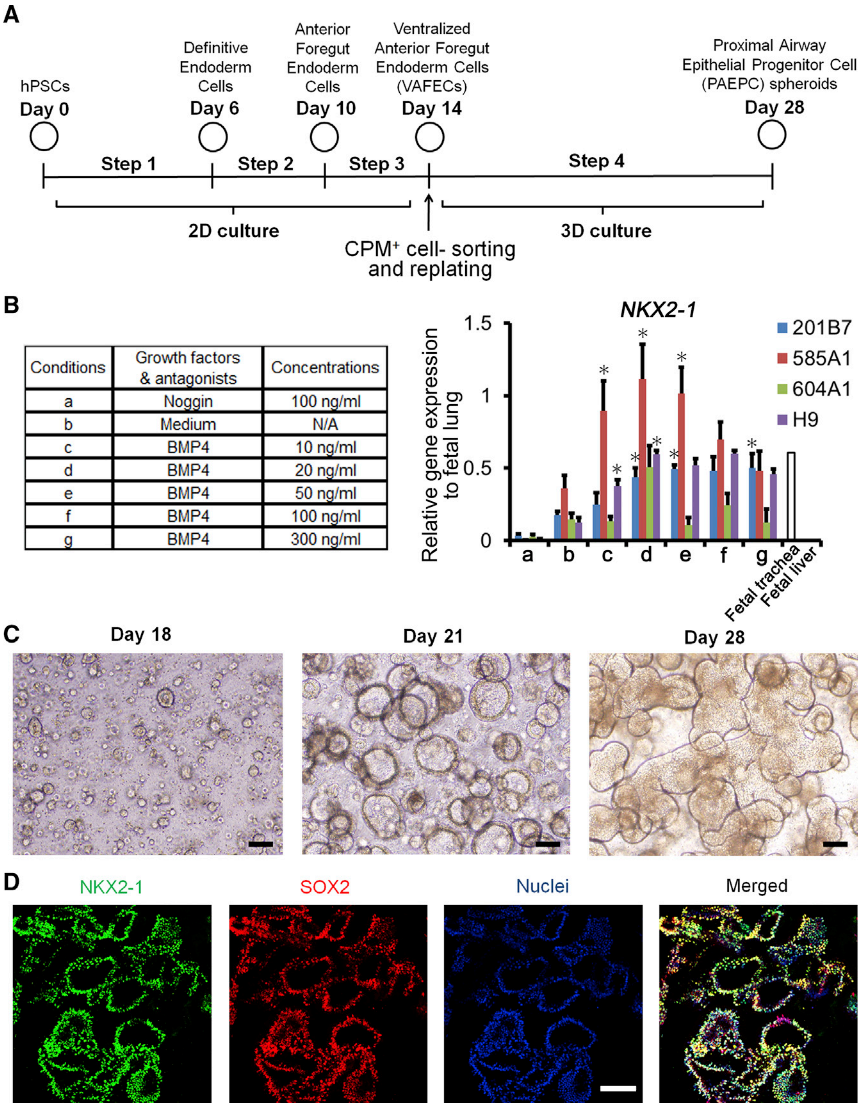

Figure 1. Generation of PAEPC Spheroids from CPM^{+} VAFECs in 3D Culture

(A) Stepwise differentiation to PAEPC spheroids from hPSCs.

(B) qRT-PCR of *NKX2-1* expression in each hPSC line on day 14, according to the dose of BMP4. The concentrations of BMP4 for each condition in Step 3 are shown in the columns. Each value was normalized to *β-ACTIN*. The gene expression level of the fetal lungs was set at 1. Error bars represent the mean ± SEM (n = 3 independent experiments). Each condition was compared with condition b for each hPSC line; $*p < 0.05$. N/A, not applicable.

(C) CPM^{+} VAFEC-derived spheroids (201B7 hiPSCs) on days 18, 21, and 28.

(D) CIF imaging shows 201B7 hiPSC-derived PAEPC spheroids coexpressing SOX2 and NKX2-1 on day 28.

Scale bars, 100 μm. See also Figure S1 and Tables S1 and S2.

28 (Figures S1B and S1C), and the medium condition of 3 μM CHIR99021 and 100 ng/ml FGF10 was chosen. Under all conditions, *SOX9* was only slightly detected by qRT-PCR (Figure S1C). In Step 4, the spheroids grew larger and some of them began to fuse by day 28 (Figure 1C). Importantly, confocal immunofluorescence (CIF) imaging studies showed that nearly all the cells forming spheroids were SOX2^{+}NKX2-1^{+} cells (Figure 1D), whereas SOX9 was not detected (data not shown), indicating that these cells were of PAEC lineage (Que et al., 2009).

Derivation of PAECs from PAEPC Spheroids

At the end of Step 4, no MCACs were observed, which prompted us to hypothesize that there might be another step for inducing MCACs. Therefore, we switched the medium to Step 5 medium based on PneumaCult-ALI medium (P-ALI) (Stemcell Technologies), a medium for primary bronchial epithelial cells (Figure 2A). On day 42, clusters of MCACs were observed by H&E staining (Figure 2B). CIF imaging revealed acetylated tubulin (Ac-Tub)$^{+}$FOXJ1^{+} cells and closely aligned Ac-Tub^{+} cells and MUC5AC^{+} cells, as observed in the fetal human lung (FHL), while secreted MUC5AC markedly accumulated in the closed lumen of the hPSC-derived spheroids (Figure 2C). A small number of SCGB1A1^{+}cells (club cells), KRT5^{+}cells (basal cells) and chromogranin A (CHGA)$^{+}$ and synaptophysin (SYP)$^{+}$cells (PNECs) were also found (Figure 2D). Nearly all the hPSC-derived PAECs expressed NKX2-1 (Figures 2D and S2A), consistent with the previous reports (Bilodeau et al., 2014) and CIF imaging of the FHL (Figure S2A). By triple immunostaining, each representative marker of MCACs, club cells and basal cells was expressed in the different cells

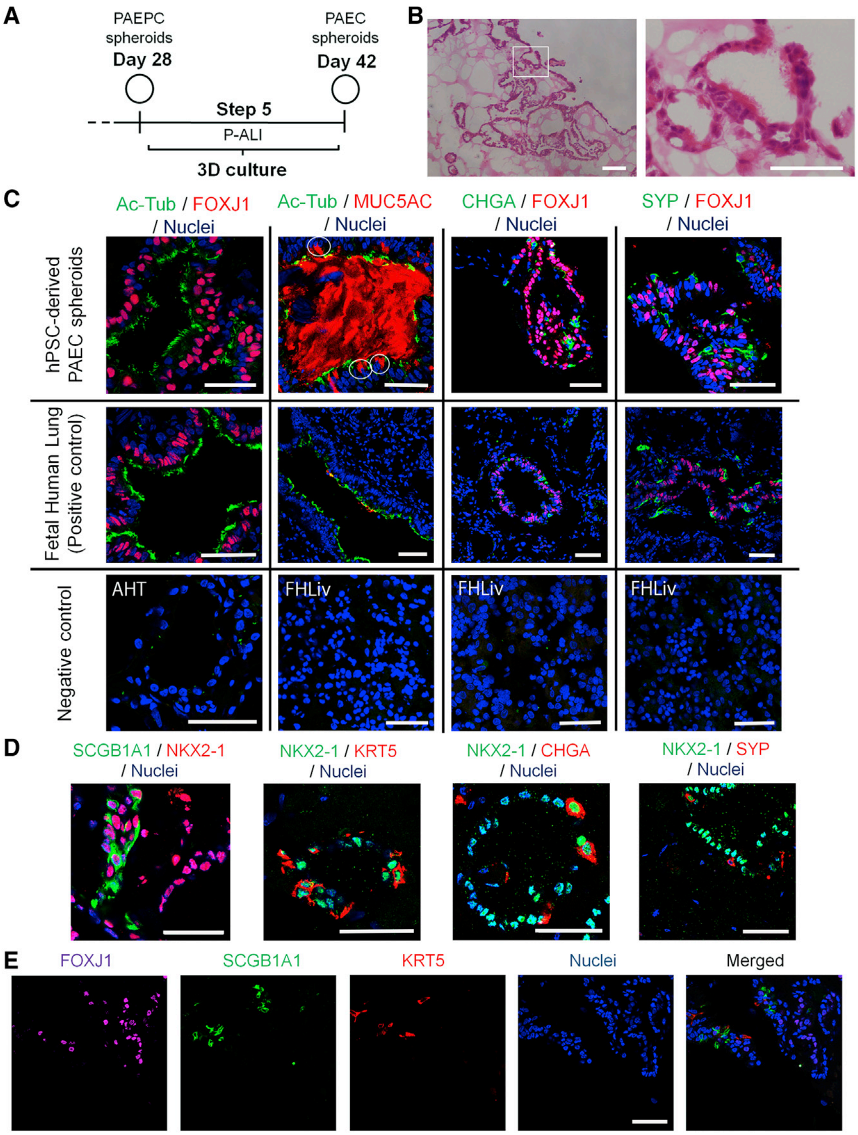

Figure 2. Derivation of PAECs from PAEPC Spheroids

(A) A schematic illustration of the induction of PAECs from hPSC-derived PAEPC spheroids.

(B) H&E staining of PAEC spheroids (201B7 hiPSCs) on day 42 shows spheroids (left) and clusters of MCACs in a magnified view (right).

(C) Double immunostaining of PAEC spheroids (201B7 hiPSCs) on day 42. Fetal human lung was shown as a positive control. Adult human thyroid (AHT) was shown as a negative control for Ac-Tub and FOXJ1, whereas fetal human liver (FHLiv) was shown as a negative control for Ac-Tub, FOXJ1, MUC5AC, SYP, and CHGA. The white circle indicates MUC5AC$^+$ cells.

(D) Double immunostaining of induced PAEC spheroids (201B7 hiPSCs) on day 42. SCGB1A1, KRT5, CHGA, and SYP were detected in NKX2-1$^+$ cells.

(E) Triple immunostaining of PAEC spheroids (201B7 hiPSCs) on day 42. FOXJ1, SCGB1A1, and KRT5 were detected in the different cells.

Scale bars, 50 μm. See also Figure S2 and Table S2.

(Figure 2E). FOXJ1$^+$ cells did not overlap with the CHGA$^+$ or SYP$^+$ cells as in the FHL (Figure 2C). PGP9.5, another PNEC marker (Linnoila, 2006), was confirmed to be expressed in both CHGA$^+$ and SYP$^+$ cells (Figures S2B and S2C).

DAPT Leads to the Efficient Induction of MCACs and Increases PNECs

Because FOXJ1 is reportedly expressed before multi-ciliogenesis in vitro and in vivo (You et al., 2004; Rawlins et al., 2007), *SNTN*, which specifically marks MCACs (Kubo et al., 2008), was adopted to detect the suitable conditions for multi-ciliogenesis. *SNTN* was significantly increased on day 42 (Figure 3A, condition b), compared with day 28 (Figure 3A, condition a) in all hPSC lines ($p < 0.05$) (Figure 3B). In addition, each PAEC marker of MCACs (Figures 3B and S3B), club cells (Figures 3C and S3C), PNECs (Figures 3D and S3D), basal cells (Figure S3E), and mucus-producing cells (Figures 3E and S3F) increased after starting 3D culture (Figure S3A, condition a, b or both) compared with before 3D culture (days 6 and 14), while *AQP5* and *SFTPC* (alveolar type I and II cells, respectively) were almost negative (Figure S3G). *SFTPB* only slightly increased in accordance with an elevation of club cell markers (Figures 3C, S3C, and S3G, protocols a and b). *PAX6* (neuronal cells) and *PAX8* (thyroid cells) were also negative (data not shown). Next, the 3D protocol (Figure S3A, protocol a) was compared with the two-dimensional protocol (Figure S3A, protocol f) between days 14 and 28, resulting in an increase of some PAEC markers (Figures S3C, S3D, and S3F, protocols a and f). Because the cells

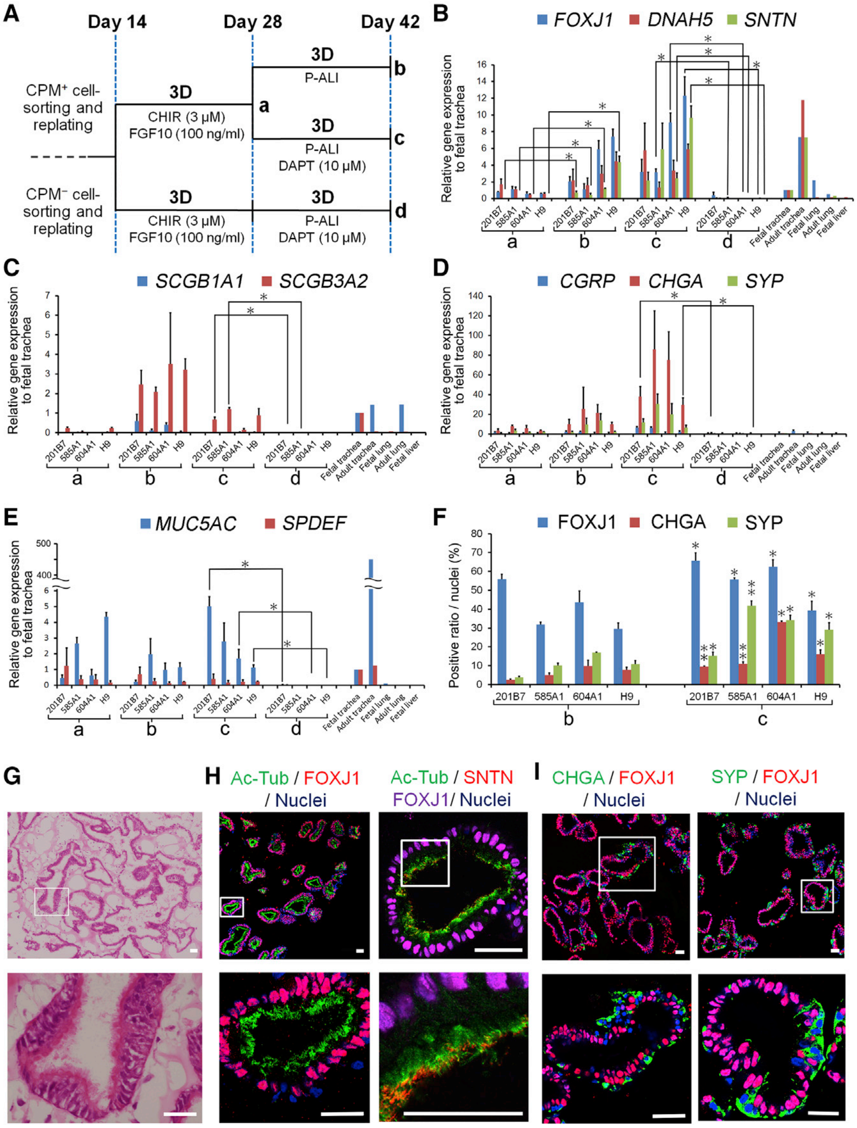

Figure 3. Directed Induction of MCACs and PNECs by Adding DAPT

(A) A schematic illustration of each protocol for induction of PAECs, according to CPM-based sorting and the addition of DAPT.

(B–E) qRT-PCR of representative PAEC markers: *FOXJ1*, *DNAH5*, and *SNTN* for MCACs (B), *SCGB1A1* and *SCGB3A2* for club cells (C), *CGRP*, *CHGA*, and *SYP* for PNECs (D), and *MUC5AC* and *SPDEF* for mucus-producing cells (E). Each value was normalized to *β-ACTIN*. The gene expression of the fetal trachea sample was set at 1. Error bars represent the mean ± SEM (n = 3 independent experiments; *p < 0.05).

(F) The induction efficiency of MCACs and PNECs calculated by counting the number of FOXJ1$^+$, CHGA$^+$, and SYP$^+$ cells (Supplemental Experimental Procedures). Error bars represent the mean ± SEM (n = 3 independent experiments). Protocol c was compared with protocol b for each hPSC line; *p < 0.05, **p < 0.01.

(G) H&E staining of DAPT-induced 3D spheroids (201B7 hiPSCs) on day 56 (upper panel) showed consecutively aligned MCACs in a magnified view (lower panel).

(H) Double and triple immunostaining of MCAC markers in DAPT-induced PAEC spheroids (201B7 hiPSCs) on day 56. Magnified views were shown in lower panels.

(I) Double immunostaining of CHGA or SYP (PNEC markers) with FOXJ1 in DAPT-induced PAEC spheroids (201B7 hiPSCs) on day 56. None of the markers was expressed in FOXJ1$^+$ cells.

Scale bars, 25 μm. See also Figure S3 and Tables S1 and S2.

spontaneously detached in 3D culture after day 28, three 3D protocols after the induction of VAFECs (Figure S3A, protocols b, c, and e) were compared with the four air-liquid interface (ALI) protocols (Figure S3A, protocols g–j), which involved two protocols modified from previous reports (Figure S3A, protocols i and j) (Wong et al., 2012; Firth et al., 2014; Supplemental Experimental Procedures).

DAPT was added to the media from days 28 to 42 (Figures 3A and S3A, protocol c and d) to increase hPSC-derived FOXJ1$^+$ cells (Figure 3F). On day 42, the 3D protocols for CPM$^+$ cells (Figures 3A and S3A, protocols b and c) appeared to induce higher gene expressions of MCAC and club cell markers than the 3D protocol for CPM$^-$ cells (Figures 3A and S3A, protocol d) and ALI protocols (Figure S3A, protocols g–j), while the 3D protocol for CPM$^-$ cells (Figures 3A and S3A protocol d) appeared to induce *KRT5* (a marker of both airway and esophageal basal cells), but not *NKX2-1* (Figure S3E). Importantly, *SNTN* increased only in the 3D protocols for CPM$^+$ cells (Figures 3A and S3A, protocols b and c). Therefore, we concluded that the 3D protocols for CPM$^+$ cells were beneficial for the induction of PAECs.

Next, we extended the culture period to day 56 (Figure S3A, protocol e), which increased *FOXJ1*, *DNAH5*, and *SNTN*. H&E staining and CIF imaging revealed that MCACs comprised a major part of the epithelia (Figures 3G and 3H). The rate of hPSC-derived FOXJ1$^+$ cells was quantified on day 56 and compared with that on day 42, resulting in an increase in the ratio of FOXJ1$^+$ cells to the total number of cells up to 85.65 ± 1.59% (p = 0.043), 85.82 ± 3.35% (p = 0.030), 72.7 ± 6.6% (p = 0.105), and 87.06 ± 0.43%

($p = 0.011$) in each hPSC line of 201B7, 585A1 and 604A1 hiPSCs, and H9 hESCs, respectively ($n = 3$ independent experiments). SNTN was localized at the tips of multiple cilia on day 56 (Figure 3H), which is consistent with the qRT-PCR results (Figures 3B and S3B). Moreover, hPSC-derived $CHGA^+$ cells and SYP^+ cells on day 42 (Figure 3A, protocols b and c) increased by adding DAPT (Figure 3F), consistent with the qRT-PCR results (Figures 3D and S3D, protocols b and c). Both the $CHGA^+$ and SYP^+ cells were localized to the aligning epithelium sparing $FOXJ1^+$ cells (Figure 3I). In all the comparisons, the vehicle control (DMSO) was added to the media under the counterpart conditions in order to exclude the effects by DMSO solvent of DAPT.

DAPT Suppresses the Notch Pathway in hPSC-Derived PNECs and Induces Functional Motile Cilia in hPSC-Derived MCACs

To elucidate the role of the Notch pathway in DAPT-induced differentiation of PNECs, NOTCH1 intracellular domain (N1ICD), HES1, and PGP9.5 were triply immunostained on day 42, and $N1ICD^+HES1^+$ cells were detected among the small number of non-PNECs (Figure S4A). By qRT-PCR, *DLL1* was significantly upregulated by DAPT in the H9 hESC line ($p = 0.002$), but not significantly in 201B7, 585A1, and 604A1 hiPSC lines ($p = 0.114$, 0.128, and 0.215, respectively). *HES1* was significantly suppressed by DAPT in the H9 hESC line ($p = 0.013$), but not significantly in 201B7, 585A1, and 604A1 hiPSC lines ($p = 0.063$, 0.225, and 0.44, respectively). *NOTCH1-3* on day 42 were unaffected, compatible with DAPT-mediated suppression of the Notch pathway (Figure S4B).

Next, the morphology of hPSC-derived MCACs was examined using electron microscopy, demonstrating multiple cilia originating from individual basal bodies on the apical surface of columnar epithelial cells (Figures 4A and S4C) and a "9+2" structure consisting of nine doublet and a central pair of singlet microtubules with dynein arms (Figure 4A, rightmost panel), which are specific features of motile cilia (Gibbons and Rowe, 1965).

On light microscopy, beating cilia were easily observed in the lumen of the spheroids and recorded by a high-speed camera (Movie S1, left panel). Metachronal wave-like beating of the cilia (Machemer, 1972) was observed in some MCACs (Figure S4D). In order to quantify the mucociliary flow over the MCACs, we established a protocol of passaging hPSC-derived MCACs in PAEC spheroids to ALI condition (Figure 4B, 3D-ALI protocol) due to the difficulty in measuring the flow rate inside the 3D spheroids. On day 56 of the 3D-ALI protocol, ciliary beating was observed on the apical side of MCACs (Movie S1, right panel). SNTN was localized at the tips of multiple cilia (Figure 4C), and CFTR was detected in the apical surface of MCACs (Figure S4E) and *FOXJ1*, *DNAH5*, *SNTN*, and *CFTR* levels appeared to be slightly, but not significantly, lower in the 3D-ALI protocol than in the 3D protocol (Figure 4D).

The ciliary beating frequency (CBF) was calculated by acquiring bright-field images of MCACs in the spheroids and the 3D-ALI condition based on the concepts previously described (Sisson et al., 2003) (Figure S4F; Supplemental Experimental Procedures). The CBF of each hiPSC line (201B7, 585A1, and 604A1)-derived MCACs showed 8.9 ± 0.27, 9.3 ± 0.34, and 6.5 ± 0.17 Hz in the spheroids on day 42 and 10.9 ± 0.31, 10.5 ± 0.26, and 10.0 ± 0.17 Hz in the 3D-ALI condition, respectively. A similar CBF was calculated for normal human bronchial epithelial cell (NHBEC)-derived MCACs in each condition (8.7 ± 0.30 Hz in the spheroids and 8.1 ± 0.33 Hz in the ALI condition) (Figure 4E). To measure mucociliary transport, the fluorescent beads placed on MCACs were traced (Movie S2; Figure 4F; Supplemental Experimental Procedures). The estimated flow velocity of the beads was approximately 7.4–10.1 μm/s in both hPSC- and NHBEC-derived MCACs. However, the values appeared to be affected by the lack of synchroneity of ciliary beating for generating a unidirectional flow (Movie S2; Figure 4F). Therefore, we analyzed the diffusion of the beads from their trajectories based on the concepts in a previous report (Qian et al., 1991). We defined the diffusion coefficient normalized to Brownian motion as the mucociliary transport index (MTI) (Supplemental Experimental Procedures). Then, the MTIs in the hPSC- and NHBEC-derived MCACs were calculated, and all the hPSC-derived MCACs showed slightly smaller MTIs compared with NHBEC-derived MCACs and significantly greater MTIs compared with Brownian motion (Figure 4G).

DISCUSSION

We established a method of 3D differentiation without feeder cells to generate hPSC-derived PAEC spheroids via isolated progenitor cells using CPM as a surface antigen, which is reportedly a biomarker of lung diseases, such as acute pneumonia and lung cancer (Dragović et al., 1995). It is noted that the inhibition of the Notch pathway induced not only MCACs but also PNECs from hPSCs, which is consistent with the studies of genetic murine models (Tsao et al., 2009; Morimoto et al., 2012). PNECs have been proposed to be the origin of small-cell lung cancer (Song et al., 2012), thus suggesting its future application in cancer studies.

The ciliary function analyses of hPSC-derived MCACs, as well as induction efficiency, are important aspects of the present study. Previously, the functional analyses of hPSC-derived PAECs mostly focused on CFTR (Wong et al., 2012; Firth et al., 2014), and not on ciliary

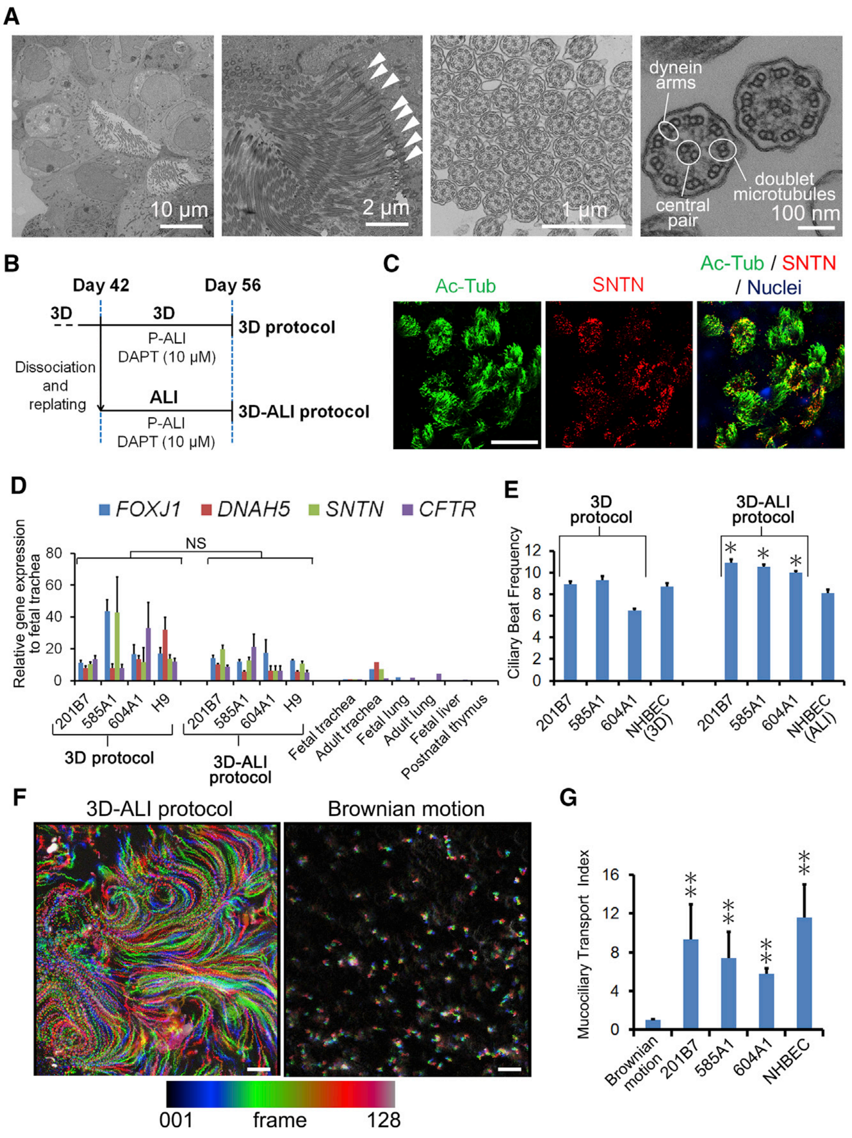

Figure 4. Characterization of Motile Cilia of hPSC-Derived MCACs

(A) Transmission electron microscopy of DAPT-induced PAEC spheroids (201B7 hiPSCs) on day 56 (leftmost panel). Multiple cilia were originated from basal bodies (second left panel, arrowheads). A cross-sectional image of multiple cilia of induced MCACs (second right panel) was magnified to show the "9+2" structure with dynein arms (rightmost panel, circles).

(B) A schematic illustration of the preparation of hPSC-derived MCACs for ciliary function tests. In the "3D protocol," MCACs were differentiated until day 56 in spheroids. In the "3D-ALI protocol," PAECs were dissociated from 3D Matrigel blocks on day 42, followed by replating and culturing under ALI condition until day 56.

(C) Double immunostaining of Ac-Tub and SNTN in 201B7 hiPSC-derived MCACs cultured in the 3D-ALI protocol.

(D) qRT-PCR of *FOXJ1*, *DNAH5*, *SNTN*, and *CFTR* expression in hPSC-derived MCACs cultured in the 3D and 3D-ALI protocols (n = 3 independent experiments). Each value was normalized to *β-ACTIN*. The gene expression of the fetal trachea sample was set at 1. NS, not significant.

(E) The CBF of MCACs in spheroids and 3D-ALI condition in each hiPSC line in three independent experiments (n = 198, 135, and 314 cells in the spheroids and n = 174, 236, and 519 cells in the 3D-ALI condition derived from 201B7, 585A1, and 604A1 hiPSCs, respectively). NHBEC-derived MCACs were used as positive controls in each condition in three independent experiments (n = 123 and 86 in the 3D and the ALI condition, respectively). The CBF in the spheroids was compared with that in the 3D-ALI condition for each hiPSC line; $^*p < 0.05$.

(F) Stacked images of the fluorescent beads placed on 201B7 hiPSC-derived MCACs (left panel), and Brownian motion (right panel) acquired for 14.2 s. Color spectrum reflected time course.

(G) The MTI of MCACs calculated from >100 trajectories of the fluorescent beads per sample in each hiPSC line in three independent experiments (n = 109, 142, 174, and 147 trajectories in 201B7, 585A1, and 604A1 hiPSCs and NHBECs, respectively). The MTIs in hiPSC-derived MCACs were compared with Brownian motion (n = 50) for each hiPSC line; $^{**}p < 0.01$. Error bars in the qRT-PCR, CBF, and MTI analyses represent the mean ± SEM.

Scale bars, 25 μm unless otherwise indicated. See also Figure S4; Movies S1 and S2; and Tables S1 and S2.

movement. In addition, the ciliary function was not shown in hPSC-derived lung organoids due to immaturity (Dye et al., 2015). In the ciliary function tests, the CBF of hPSC-derived MCACs in spheroids appeared to be lower than that in the 3D-ALI protocol (Figure 4E) for at least two reasons. First, mucoid secretion was trapped in the closed lumen and its increased viscosity might reduce the CBF in the spheroids (Figure 2C and Movie S1, left). Second, we had to mince the spheroids and place cover slips on the samples during image acquisition, which may have reduced the CBF in the hPSC-derived spheroids, while we could directly observe the samples in the 3D-ALI protocol. The CBF of hPSC-derived MCACs in the 3D-ALI protocol was near the normal CBF of human MCACs, which range from 10 to 14 Hz (Rutland et al., 1982). Next, to quantify mucociliary transport, fluorescent beads were

tracked as previously demonstrated in resected murine trachea (Kunimoto et al., 2012). Because synchronized ciliary beating for generating a unidirectional flow appeared to be incomplete in both hPSC- and NHBEC-derived MCACs (Movies S1 and S2; Figure 4F), as was reported for NHBEC-derived MCACs (Matsui et al., 1998), we further focused on the diffusion of the beads, demonstrating the potency of mucociliary clearance in hPSC-derived MCACs (Figures 4F and 4G). The difference between MCACs derived from hPSCs and NHBECs might be partly due to the difference in maturity. In addition, the ideal balance in the number of between MCACs and mucus-producing cells for mucociliary clearance remains to be elucidated. *MUC5AC* and *SPDEF* levels on day 56 were lower than on day 42 (Figure S3F), which might be due to differentiation (Chen et al., 2009) and/or apoptosis. In this respect, the regulation of $MUC5AC^+$ cells by modulating factors, such as IL-13 (Atherton et al., 2003), remains to be a future subject. In conclusion, the findings of the present study are thus considered to pave the way for future applications toward modeling airway diseases, such as PCD and CF, or developing methods of airway reconstruction such as an artificial trachea.

EXPERIMENTAL PROCEDURES

Imaging for the CBF and MTI

To measure the CBF, movies of hPSC- and NHBEC-derived MCACs were captured on a high-speed camera (FASTCAM MC2.1; Photron) connected to an upright microscope (Zeiss Axioplan; Carl Zeiss) with ×63 objectives. To measure MTI, the flow of fluorescent beads (Fluoresbrite, 0.5 µm; Polysciences) was recorded by an Orca-ER CCD camera (Hamamatsu) connected to an upright fluorescent microscope (BX51; Olympus) with a ×20 objective. See the Supplemental Experimental Procedures.

Ethics

The use of H9 hESCs was approved by the Ministry of Education, Culture, Sports, Science, and Technology (MEXT) of Japan. For the use of human samples, human ethics approval was obtained from the Institutional Review Board and Ethics Committee of Kyoto University Graduate School and Faculty of Medicine.

Statistical Analysis

At least three independent experiments were conducted in each study. The values are expressed as the means ± SEM. A two-tailed t test was performed to determine the statistical significance. $p < 0.05$ was considered to be significant.

SUPPLEMENTAL INFORMATION

Supplemental Information includes Supplemental Experimental Procedures, four figures, two tables, and two movies and can be found with this article online at http://dx.doi.org/10.1016/j.stemcr.2015.11.010.

AUTHOR CONTRIBUTIONS

S.G., S.K., K.T., and S.T. designed the study. S.K., K.T., S.G., Y.Y., Y.K., and T.N., performed the experiments. S.G., S.K., K.T., T.N., and S.T. analyzed the data. S.G. and S.K. wrote the manuscript through a fruitful discussion with and supervision by K.T., H.M., S.M., T.H., I.I., S.T., and M.M.

ACKNOWLEDGMENTS

We are grateful to K. Osafune, K. Okita, K. Takahashi, I. Asaka, and S. Yamanaka (Center for iPS Cell Research and Application, Kyoto University) for providing the iPSC lines, and method of iPSC culture. We thank K. Okamoto-Furuta and H. Kohda (Division of Electron Microscopic Study, Center for Anatomical Studies, Kyoto University), Y. Maeda and A. Inazumi for technical assistance. The fluorescence studies were performed at the Medical Research Support Center, Kyoto University. This work was supported by Grant-in-Aid for Scientific Research (KAKENHI), Translational Research Network Program from MEXT of Japan, Kyoto-Funding for Innovation in Health-related R&D Fields of Kyoto City, Core Research for Evolutional Science and Technology (CREST) of the Japan Science and Technology Agency (S.T.), and, in part, Uehara Memorial Foundation.

Received: April 29, 2015
Revised: November 18, 2015
Accepted: November 18, 2015
Published: December 24, 2015

REFERENCES

Atherton, H.C., Jones, G., and Danahay, H. (2003). IL-13-induced changes in the goblet cell density of human bronchial epithelial cell cultures: MAP kinase and phosphatidylinositol 3-kinase regulation. Am. J. Physiol. Lung Cell. Mol. Physiol. *285*, L730–L739.

Bilodeau, M., Shojaie, S., Ackerley, C., Post, M., and Rossant, J. (2014). Identification of a proximal progenitor population from murine fetal lungs with clonogenic and multilineage differentiation potential. Stem Cell Rep. *3*, 634–649.

Chen, G., Korfhagen, T.R., Xu, Y., Kitzmiller, J., Wert, S.E., Maeda, Y., Gregorieff, A., Clevers, H., and Whitsett, J.A. (2009). SPDEF is required for mouse pulmonary goblet cell differentiation and regulates a network of genes associated with mucus production. J. Clin. Invest. *119*, 2914–2924.

Dragović, T., Schraufnagel, D.E., Becker, R.P., Sekosan, M., Votta-Velis, E.G., and Erdös, E.G. (1995). Carboxypeptidase M activity is increased in bronchoalveolar lavage in human lung disease. Am. J. Respir. Crit. Care Med. *152*, 760–764.

Dye, B.R., Hill, D.R., Ferguson, M.A.H., Tsai, Y.H., Nagy, M.S., Dyal, R., Wells, J.M., Mayhew, C.N., Nattiv, R., Klein, O.D., et al. (2015). In vitro generation of human pluripotent stem cell derived lung organoids. Elife *4*, e05098.

Firth, A.L., Dargitz, C.T., Qualls, S.J., Menon, T., Wright, R., Singer, O., Gage, F.H., Khanna, A., and Verma, I.M. (2014). Generation of multiciliated cells in functional airway epithelia from human induced pluripotent stem cells. Proc. Natl. Acad. Sci. USA *111*, E1723–E1730.

Gibbons, I.R., and Rowe, A.J. (1965). Dynein: a protein with adenosine triphosphatase activity from cilia. Science *149*, 424–426.

Gotoh, S., Ito, I., Nagasaki, T., Yamamoto, Y., Konishi, S., Korogi, Y., Matsumoto, H., Muro, S., Hirai, T., Ogawa, S., et al. (2014). Generation of alveolar epithelial spheroids via isolated progenitor cells from human pluripotent stem cells. Stem Cell Rep. *3*, 394–403.

Huang, S.X., Islam, M.N., O'Neill, J., Hu, Z., Yang, Y.G., Chen, Y.W., Mumau, M., Green, M.D., Vunjak-Novakovic, G., Bhattacharya, J., et al. (2014). Efficient generation of lung and airway epithelial cells from human pluripotent stem cells. Nat. Biotechnol. *32*, 84–91.

Kimura, S., Hara, Y., Pineau, T., Fernandez-Salguero, P., Fox, C.H., Ward, J.M., and Gonzalez, F.J. (1996). The T/ebp null mouse: thyroid-specific enhancer-binding protein is essential for the organogenesis of the thyroid, lung, ventral forebrain, and pituitary. Genes Dev. *10*, 60–69.

Kubo, A., Yuba-Kubo, A., Tsukita, S., Tsukita, S., and Amagai, M. (2008). Sentan: a novel specific component of the apical structure of vertebrate motile cilia. Mol. Biol. Cell. *19*, 5338–5346.

Kunimoto, K., Yamazaki, Y., Nishida, T., Shiojara, K., Ishikawa, H., Hasegawa, T., Okanoue, T., Hamada, H., Noda, T., Tamura, A., et al. (2012). Coordinated ciliary beating requires Odf2-mediated polarization of basal bodies via basal feet. Cell *148*, 189–200.

Linnoila, R.I. (2006). Functional facets of the pulmonary neuroendocrine system. Lab Invest. *86*, 425–444.

Machemer, H. (1972). Ciliary activity and the origin of metachrony in Paramecium. J. Exp. Biol. *57*, 239–259.

Matsui, H., Randell, S.H., Peretti, S.W., Davis, C.W., and Boucher, R.C. (1998). Coordinated clearance of periciliary liquid and mucus from airway surfaces. J. Clin. Invest. *102*, 1125–1131.

Morimoto, M., Nishinakamura, R., Saga, Y., and Kopan, R. (2012). Different assemblies of Notch receptors coordinate the distribution of the major bronchial Clara, ciliated and neuroendocrine cells. Development *139*, 4365–4373.

Mou, H., Zhao, R., Sherwood, R., Ahfeldt, T., Lapey, A., Sicillian, L., Izvolsky, K., Musunuru, K., Cowan, C., and Rajagopal, J. (2012). Generation of multipotent lung and airway progenitors from mouse ESCs and patient-specific cystic fibrosis iPSCs. Cell Stem Cell *10*, 385–397.

Nishimura, Y., Hamazaki, T.S., Komazaki, S., Kamimura, S., Okochi, H., and Asashima, M. (2006). Ciliated cells differentiated from mouse embryonic stem cells. Stem Cells *24*, 1381–1388.

Okita, K., Yamakawa, T., Matsumura, Y., Sato, Y., Amano, N., Watanabe, A., Goshima, N., and Yamanaka, S. (2013). An efficient nonviral method to generate integration-free human-induced pluripotent stem cells from cord blood and peripheral blood cells. Stem Cells *31*, 458–466.

Qian, H., Sheetz, M.P., and Elson, E.L. (1991). Single particle tracking. Analysis of diffusion and flow in two-dimensional systems. Biophys. J. *60*, 910–921.

Que, J., Luo, X., Schwartz, R.J., and Hogan, B.L. (2009). Multiple roles for Sox2 in the developing and adult mouse trachea. Development *136*, 1899–1907.

Rawlins, E.L., Ostrowski, L.E., Randell, S.H., and Hogan, B.L. (2007). Lung development and repair: contribution of the ciliated lineage. Proc. Natl. Acad. Sci. USA *104*, 410–417.

Rock, J.R., Onaitis, M.W., Rawlins, E.L., Lu, Y., Clark, C.P., Xue, Y., Randell, S.H., and Hogan, B.L. (2009). Basal cells as stem cells of the mouse trachea and human airway epithelium. Proc. Natl. Acad. Sci. USA *106*, 12771–12775.

Rossman, C.M., Forrest, J.B., Lee, R.M., and Newhouse, M.T. (1980). The dyskinetic cilia syndrome. Ciliary motility in immotile cilia syndrome. Chest *78*, 580–582.

Rutland, J., Griffin, W.M., and Cole, P.J. (1982). Human ciliary beat frequency in epithelium from intrathoracic and extrathoracic airways. Am. Rev. Respir. Dis. *125*, 100–105.

Shojaie, S., Ermini, L., Ackerley, C., Wang, J., Chin, S., Yeganeh, B., Bilodeau, M., Sambi, M., Rogers, I., Rossant, J., et al. (2015). Acellular lung scaffolds direct differentiation of endoderm to functional airway epithelial cells: requirement of matrix-bound HS proteoglycans. Stem Cell Rep. *4*, 419–430.

Sisson, J.H., Stoner, J.A., Ammons, B.A., and Wyatt, T.A. (2003). All-digital image capture and whole-field analysis of ciliary beat frequency. J. Microsc. *211*, 103–111.

Song, H., Yao, E., Lin, C., Gacayan, R., Chen, M.H., and Chuang, P.T. (2012). Functional characterization of pulmonary neuroendocrine cells in lung development, injury, and tumorigenesis. Proc. Natl. Acad. Sci. USA *109*, 17531–17536.

Takahashi, K., Tanabe, K., Ohnuki, M., Narita, M., Ichisaka, T., Tomoda, K., and Yamanaka, S. (2007). Induction of pluripotent stem cells from adult human fibroblasts by defined factors. Cell *131*, 861–872.

Thomson, J.A., Itskovitz-Eldor, J., Shapiro, S.S., Waknitz, M.A., Swiergiel, J.J., Marshall, V.S., and Jones, J.M. (1998). Embryonic stem cell lines derived from human blastocysts. Science *282*, 1145–1147.

Tsao, P.N., Vasconcelos, M., Izvolsky, K.I., Qian, J., Lu, J., and Cardoso, W.V. (2009). Notch signaling controls the balance of ciliated and secretory cell fates in developing airways. Development *136*, 2297–2307.

Wong, A.P., Bear, C.E., Chin, S., Pasceri, P., Thompson, T.O., Huan, L.J., Ratjen, F., Ellis, J., and Rossant, J. (2012). Directed differentiation of human pluripotent stem cells into mature airway epithelia expressing functional CFTR protein. Nat. Biotechnol. *30*, 876–882.

You, Y., Huang, T., Richer, E.J., Schmidt, J.E., Zabner, J., Borok, Z., and Brody, S.L. (2004). Role of f-box factor foxj1 in differentiation of ciliated airway epithelial cells. Am. J. Physiol. Lung Cell. Mol. Physiol. *286*, 650–657.

Zhang, L., Button, B., Gabriel, S.E., Burkett, S., Yan, Y., Skiadopoulos, M.H., Dang, Y.L., Vogel, L.N., McKay, T., Mengos, A., et al. (2009). CFTR delivery to 25% of surface epithelial cells restores normal rates of mucus transport to human cystic fibrosis airway epithelium. PLoS Biol. *7*, e1000155.

Stem Cell Reports, Volume *6*

Supplemental Information

Directed Induction of Functional Multi-ciliated Cells in Proximal Airway Epithelial Spheroids from Human Pluripotent Stem Cells

Satoshi Konishi, Shimpei Gotoh, Kazuhiro Tateishi, Yuki Yamamoto, Yohei Korogi, Tadao Nagasaki, Hisako Matsumoto, Shigeo Muro, Toyohiro Hirai, Isao Ito, Sachiko Tsukita, and Michiaki Mishima

Figure S1, related to Figure 1

A

Spheroid forming conditions in Step 4	Growth Factors / Concentration			
	CHIR99021	FGF10	KGF	DAPT
**(1)**	N/A	100 ng/ml	N/A	N/A
**(2)**	1 μM	100 ng/ml	N/A	N/A
**(3)**	3 μM	N/A	N/A	N/A
**(4)**	3 μM	10 ng/ml	N/A	N/A
**(5)**	3 μM	100 ng/ml	N/A	N/A
**(6)**	3 μM	10 ng/ml	10 ng/ml	N/A
**(7)**	3 μM	100 ng/ml	10 ng/ml	N/A
**(8)**	3 μM	100 ng/ml	N/A	10 μM
**(9)**	3 μM	100 ng/ml	N/A	50 μM

B

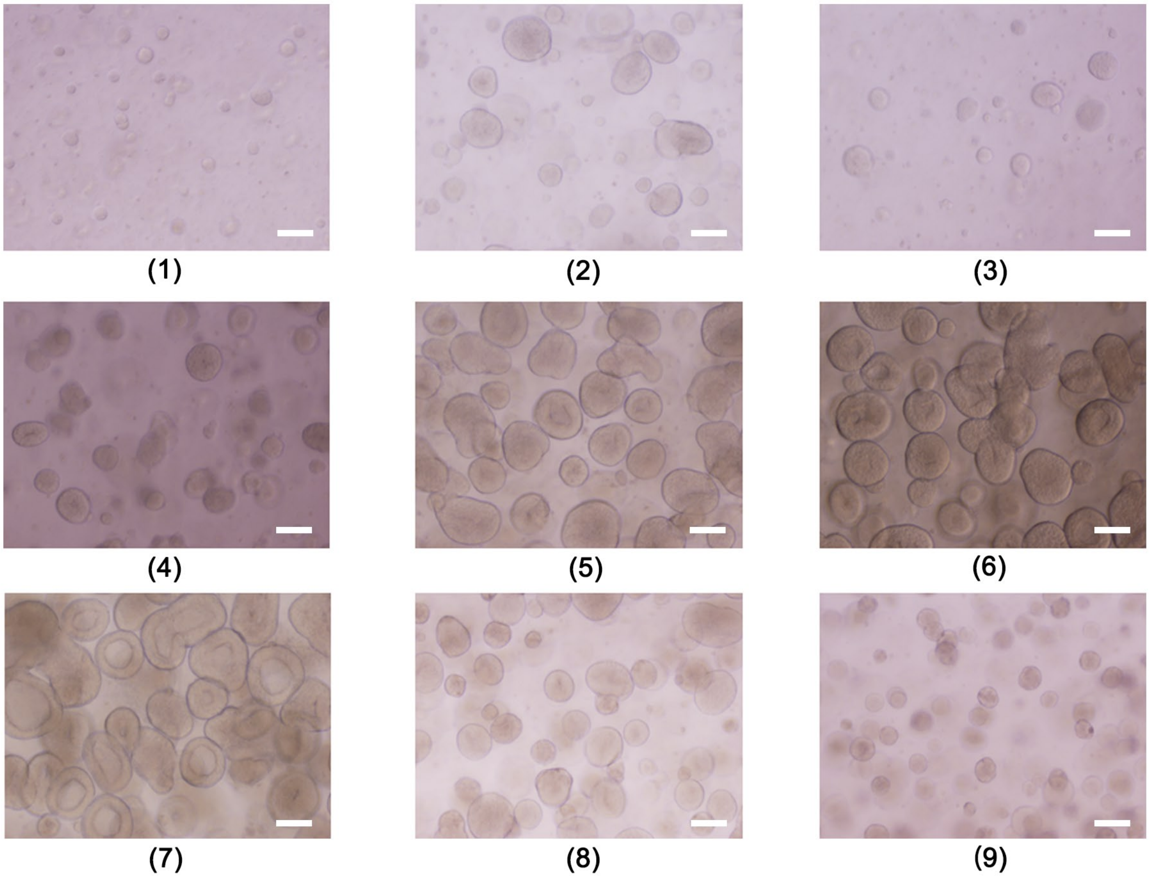

C

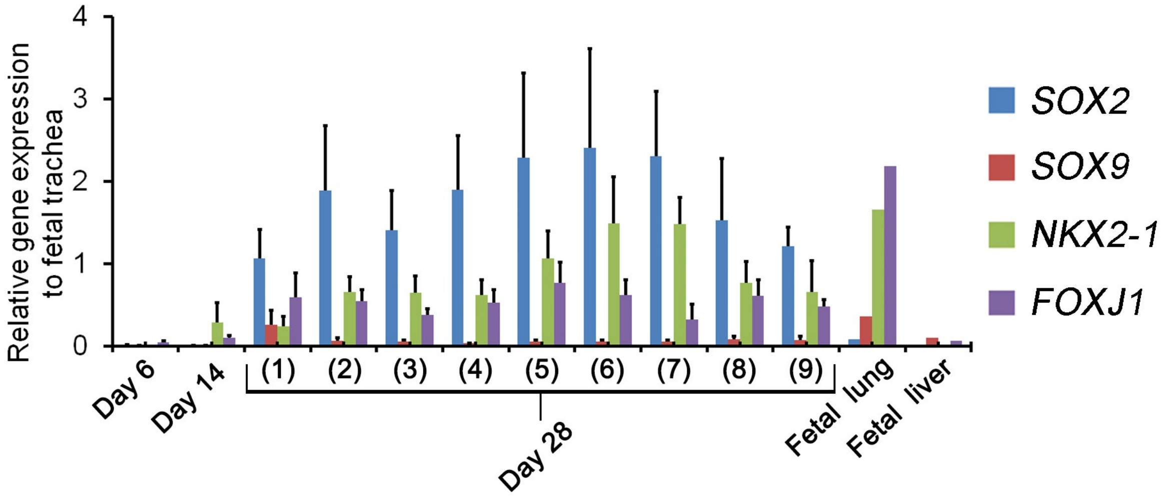

Figure S2, related to Figure 2

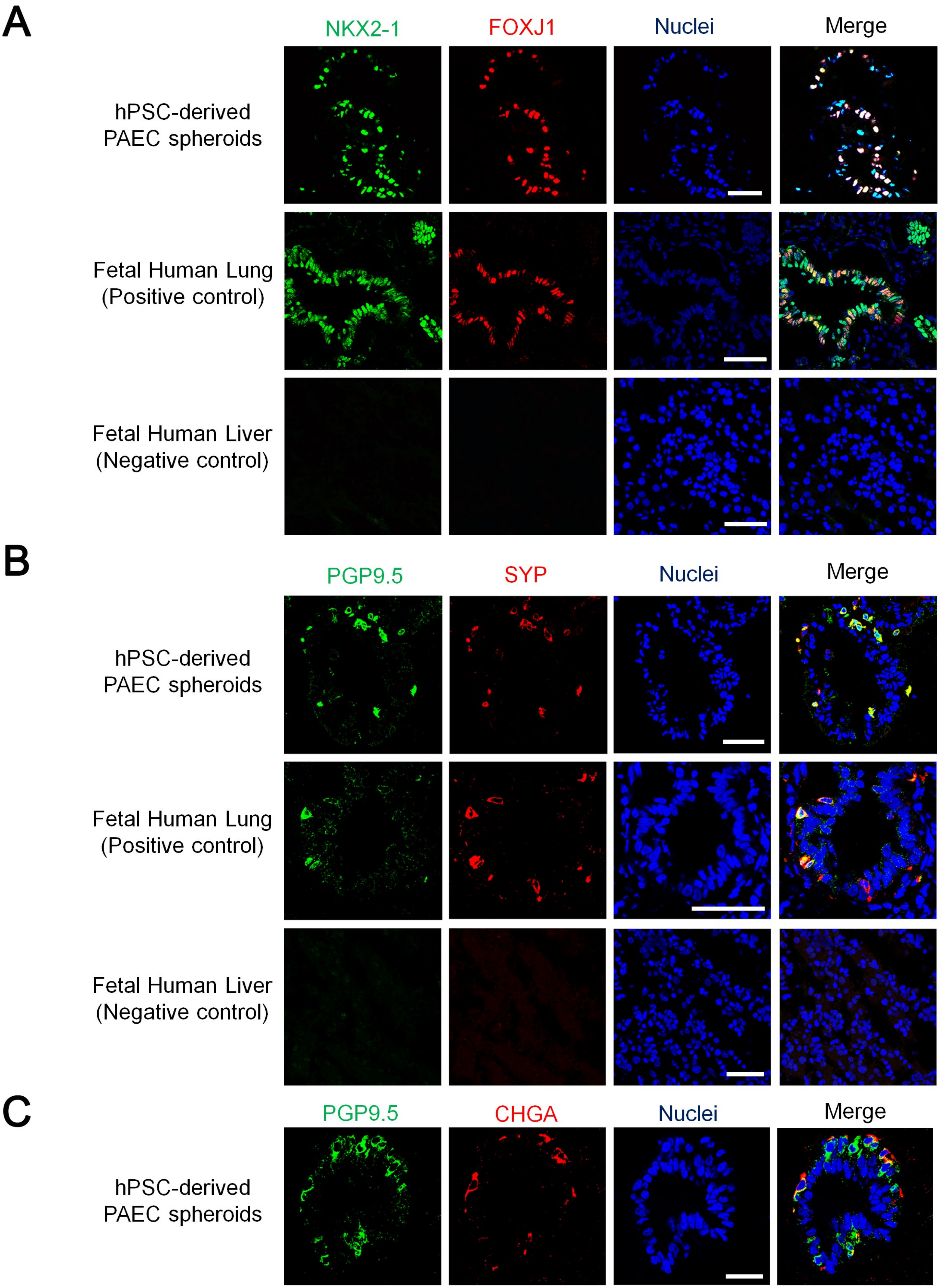

Figure S3, related to Figure 3

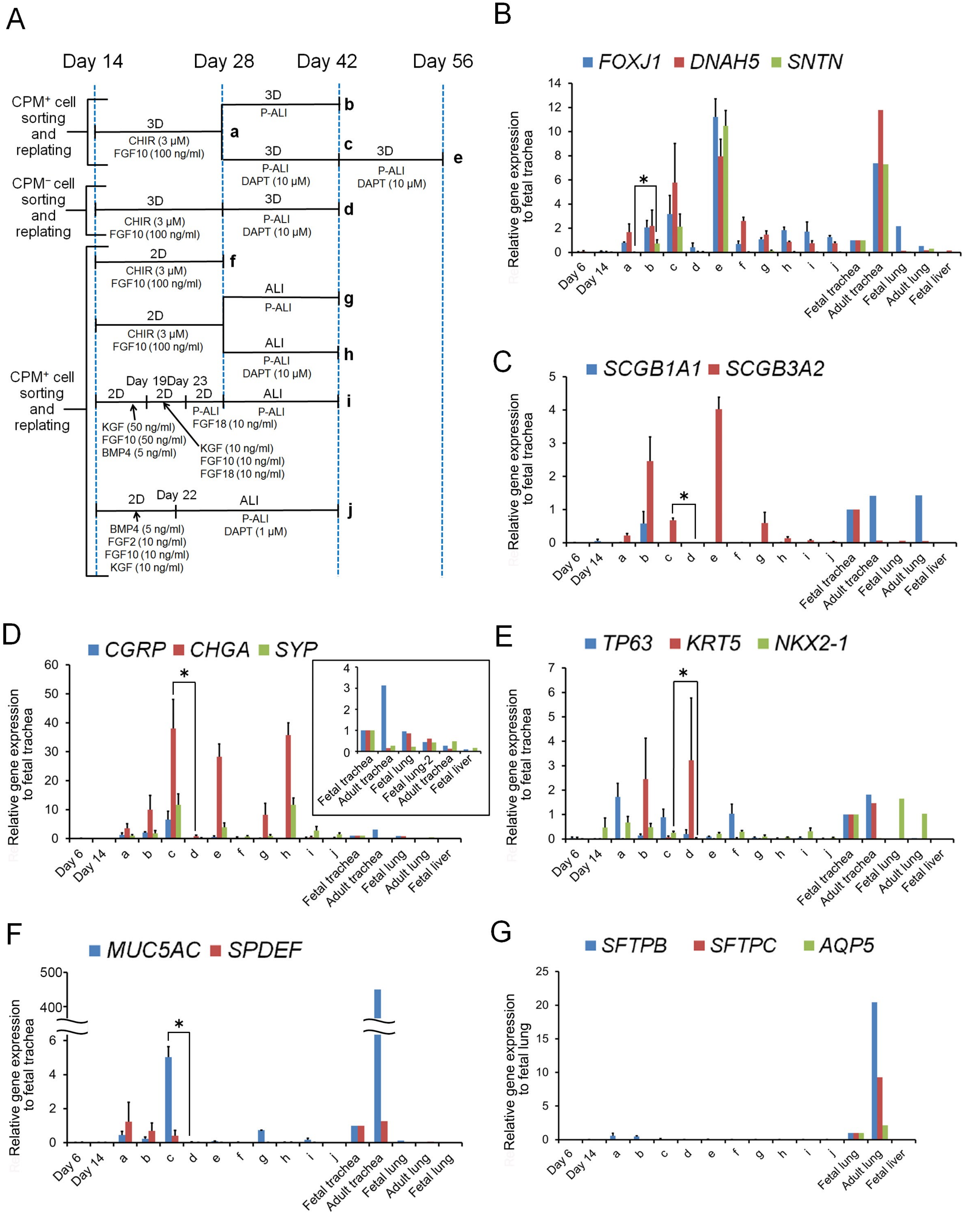

Figure S4, related to Figure 4

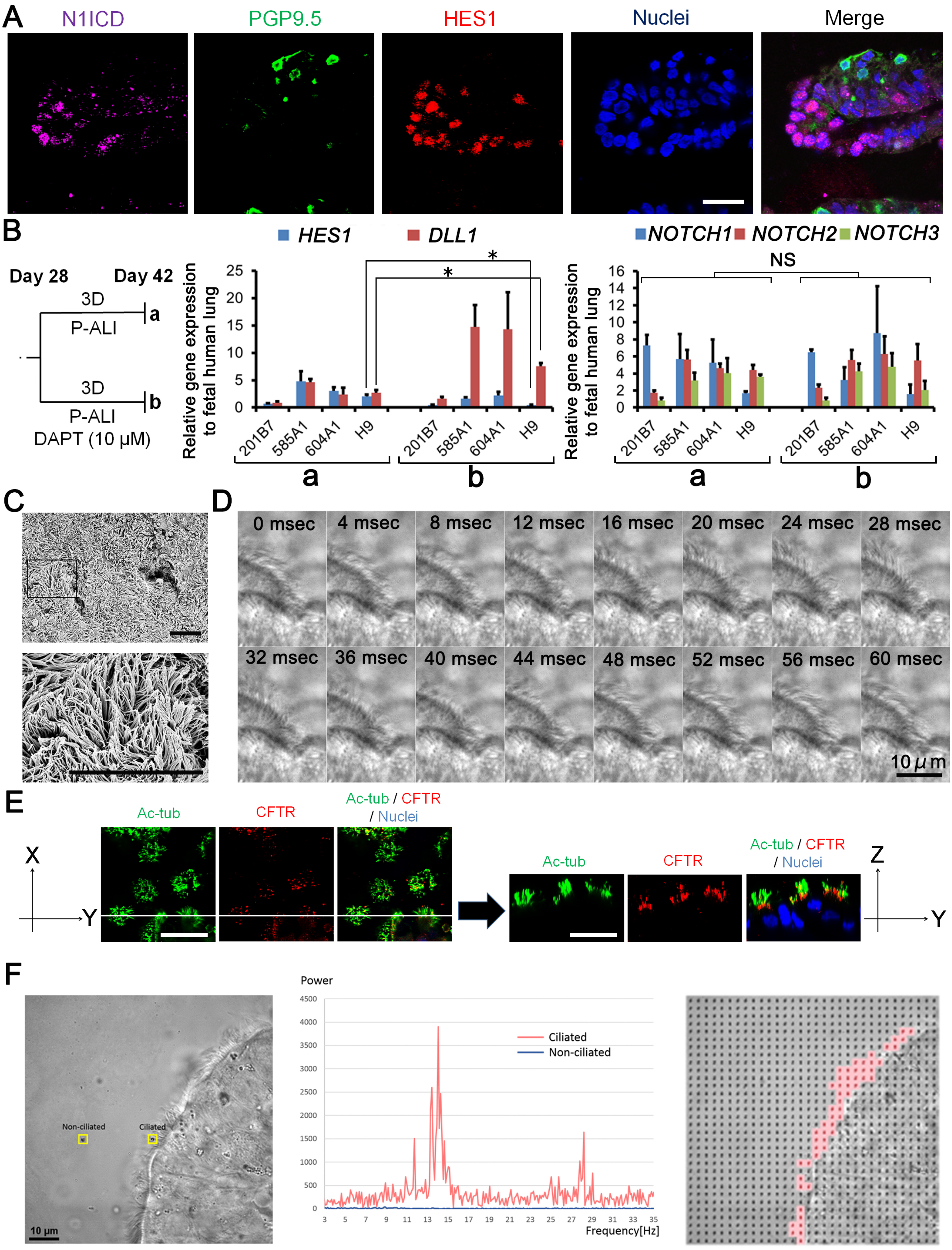

SUPPLEMENTAL FIGURE LEGENDS

Figure S1. Screening of medium conditions for forming PAEPC spheroids, related to Figure 1.

(A) Each medium condition evaluated in 3D culture in Step 4 from day 14 to day 28. (B) Representative images of the spheroids (201B7 hiPSCs) formed under each condition. Scale bars, 100 μm. (C) qRT-PCR of *SOX2*, *SOX9*, *NKX2-1* and *FOXJ1* in each condition at the end of Step 4 (day 28) (n=3 independent experiments) in 201B7 hiPSCs. Each value was normalized to *β-ACTIN*. The gene expression observed in the fetal trachea was set at 1. Error bars represent the mean ± SEM (n=3 independent experiments).

Figure S2. Characterization of hPSC-derived PAECs by immunostaining, related to Figure 2.

(A) Double immunostaining of NKX2-1 and FOXJ1 in PAEC spheroids (201B7 hiPSCs) on day 42 (top panels), fetal human lung as a positive control (middle panels), and fetal human liver as a negative control (bottom panels). Scale bars, 50 μm. (B) Double immunostaining of PGP9.5 and SYP in PAEC spheroids (585A1 hiPSCs) on day 42 (top panels), fetal human lung as a positive control (middle panels), and fetal human liver as a

negative control (bottom panels). PGP9.5 was positive in nearly all the SYP^+ cells. Scale bars, 50 μm. (C) Double immunostaining of PGP9.5 and CHGA in PAEC spheroids (585A1 hiPSCs) on day 42. Nearly all the $CHGA^+$ cells expressed PGP9.5, whereas some portion of $PGP9.5^+$ cells did not express CHGA.

Scale bars, 50 μm.

Figure S3. Comparison of gene expression of various lung lineage markers, related to Figure 3.

(A) A schematic illustration of various conditions for inducing PAECs post day 14. The same basal medium as in Steps 2 and 3 was used from days 14 to 28 of protocols a ~ i and from days 14 to 22 of protocol j. (B)~(G) qRT-PCR of each lineage cell marker in differentiated cells derived from 201B7 hiPSCs at the end of each condition (n=3 independent experiments): *FOXJ1*, *DNAH5* and *SNTN* (MCACs) (B), *SCGB1A1* and *SCGB3A2* (club cells) (C), *CGRP*, *CHGA* and *SYP* (PNECs) (D), *TP63* ,*KRT5* (basal cells), and *NKX2-1* (E), *MUC5AC* and *SPDEF* (mucus-producing cells) (F), and *SFTPB*, *SFTPC* and *AQP5* (alveolar epithelial cells) (G). Each value was normalized to *β-ACTIN*. The gene expression of the fetal human trachea sample was set at 1 in (B)~(F) and that of the fetal

human lung was set at 1 in (G). Error bars represent the mean ± SEM (n=3 independent experiments). In Figure S3D, the relative gene expressions of the positive controls (another fetal lung sample which was named as Fetal lung-2, was added) and the fetal human liver sample as a negative control were enlarged in the black square.

Figure S4. Characterization of hPSC-derived PNECs and MCACs, related to Figure 4.

(A) Triple immunostaining of PGP9.5, N1ICD and HES1 in DAPT-induced PAEC spheroids (585A1 hiPSCs) on day 42. Scale bar, 25 μm. (B) qRT-PCR of *HES1, DLL1*, *NOTCH1*, *NOTCH2* and *NOTCH3* of PAEC spheroids derived from each hPSC line on day 42. Each value was normalized to *β-ACTIN*. The gene expression level of the fetal lungs was set at 1. Error bars represent the mean ± SEM (n=3 independent experiments). NS means "not significant". $*p<0.05$. (C) Scanning electron microscopy of DAPT-induced PAEC spheroids (201B7 hiPSCs) on day 56 showed an apical surface of a spheroid covered by multiple cilia (left panel) with a magnified view (right panel). (D) Serially captured images of motile cilia of MCACs in a 3D spheroid (585A1 hiPSCs) on day 42 by a high-speed camera acquired at intervals of 4.0 ms. (E) Double immunostaining of Ac-Tub and CFTR in 201B7 hiPSC-derived PAECs cultured in the 3D-ALI protocol. Scale bar, 25

μm.

(F) A representative image of ROIs including a ciliated region or non-ciliated region in a spheroid (201B7 hiPSCs) on day 42 (left panel). Fast Fourier transform scores (FFT) obtained from the representative ROIs of the ciliated and non-ciliated regions in time-course (middle graph). ROIs involving the ciliated region were selected by FFT to calculate the CBF (pink area, right panel). Scale bar, 10 μm.

Movie S1. Ciliary beating of hiPSC-derived MCACs, related to Figure 4.

Beating cilia of MCACs in a spheroid (585A1 hiPSCs) on day 42 (left panel) and in a sheet cultured in the 3D-ALI protocol (604A1 hiPSCs) on day 56 (right panel) observed by using light microscopy and shot at 250 frames per second (fps) on a high-speed video camera.

Movie S2. Stacked images of the fluorescent beads to measure mucociliary transport, related to Figure 4.

Representative stacked images of the fluorescent beads placed on MCACs (201B7 hiPSCs) in the 3D-ALI protocol on day 56 (left panel) and Brownian motion of the beads as a negative control (right panel).

Table S1. Primers used in the present study.

Gene name		Primer Sequence	Size (bp)
β-ACTIN	F	CAATGTGGCCGAGGACTTTG	126
	R	CATTCTCCTTAGAGAGAAGTGG	
NKX2-1	F	AGGACACCATGAGGAACAGC	160
	R	GCCATGTTCTTGCTCACGTC	
SOX2	F	GCACATGAAGGAGCACCCGGATTA	86
	R	CGGGCAGCGTGTACTTATCCTTCTT	
SOX9	F	GAGGAAGTCGGTGAAGAACG	337
	R	ATCGAAGGTCTCGATGTTGG	
FOXJ1	F	CCTGTCGGCCATCTACAAGT	94
	R	AGACAGGTTGTGGCGGATT	
DNAH5	F	GCAATTGTGGCTTCCTGTTT	109
	R	GGAGACCTCCAACAGCAAAA	
SNTN	F	GCTGCAAACCCAATTTAGGA	84
	R	TGCTCATCAAGTTCAGAAAGGA	
SCGB1A1	F	CACCATGAAACTCGCTGTCAC	147
	R	AGTTCCATGGCAGCCTCATAAC	
SCGB3A2	F	CAAGTGGAACCACTGGCTTG	198
	R	CCAGAGGTAAAGGTGCCAAC	
P63	F	ACTGCCAAATTGCAAAGACA	184
	R	TGACTAGGAGGGGCAATCTG	
KRT5	F	GAGCTGAGAAACATGCAGGA	82
	R	TCTCAGCAGTGGTACGCTTG	
MUC5AC	F	CATCTGCCAGCTGATTCTGA	129
	R	AAGACGCAGCCCTCATAGAA	
SPDEF	F	AAGTGCTCAAGGACATCGAGA	94
	R	AGGAGCCACTTCTGCACATT	
CGRP	F	GGGGTGTGGTGAAGAACAAC	168
	R	CCATGGAGCCTTTCCTACAA	
CHGA	F	CGGATCCTTTCCATTCTGAG	105
	R	ACCGCTGTGTTTCTTCTGCT	
SYP	F	TTTGTGAAGGTGCTGCAATG	125
	R	ACCTCGATGCTGAGGTCACT	
SFTPB	F	GAGCCGATGACCTATGCCAAG	133
	R	AGCAGCTTCAAGGGGAGGA	
SFTPC	F	GCAAAGAGGTCCTGATGGAG	178
	R	TGTTTCTGGCTCATGTGGAG	
AQP5	F	CTGTCCATTGGCCTGTCTGTC	248
	R	GGCTCATACGTGCCTTTGATG	
PAX6	F	CGGAGTGAATCAGCTCGGTG	301
	R	CCGCTTATACTGGGCTATTTTGC	
PAX8	F	TCAACCTCCCTATGGACAGCTG	137
	R	GAGCCCATTGATGGAGTAGGTG	
CFTR	F	GATACAGACAGCGCCTGGAA	110
	R	TGAAGCCAGCTCTCTATCCCA	
HES1	F	ATGACAGTGAAGCACCTCCG	103
	R	ACTCGCTGAAGCCGGCTC	
DLL1	F	CTCCTTCAGTCTGCCCGAC	142
	R	TGTTGCGAGGTCATCAGGAG	
NOTCH1	F	GTCACCCACGAGTGTGCC	128
	R	CAGTTGTAGGTGTTCACGCC	
NOTCH2	F	ATTGCAGTGTCGAGATGGCT	141
	R	CGGTTCTTCTCACAGGGGTC	
NOTCH3	F	CAAATGGAGGTCGTTGCACC	103
	R	GAGTGACAGGGGTCCTCCA	

Table S2. Antibodies used in the present study

Primary Antibodies	Dilution rate	Manufacturer	Clone / Cat. No.
NKX2-1	1:500	Novus Biologocals	EP1584Y / NB100-80062
NKX2-1	1:500	Lab Vision	8G7G3/1 / MS-699-P
CPM	1:500	Leica microsystems	1C2 / NCL-CPMm
SOX2	1:500	EMD-Millipore	AB5603
SOX9	1:20	R&D systems	AF3075
FOXJ1	1:500	eBioscience	2A5/14-9965-82
Acetylated tubulin	1:4000	Sigma-Aldrich	T7451
SNTN	1:100	Atlas Antibodies	HPA043322
SCGB1A1	1:25	R&D systems	MAB4218/394324
KRT5	1:100	Lab Vision	EP1601Y / RM-2106-S0
MUC5AC	1:100	Thermo Scientific	45M1/MS-145-P
CHGA	1:100	Santa Cruz	sc-13090
SYP	1:100	Santa Cruz	sc-9116
CFTR	1:20	R&D systems	13-1/MAB1660
PGP9.5	1:50	abcam	13C4/ab8189
HES1	1:100	abcam	2D2/ab119776
Notch1 antibody-Cleaved-Val1744（N1ICD)	1:100	abcam	ab52301

Secondary Antibodies	Dilution rate	Manufacturer	Cat. No.
Donkey anti-mouse IgG(H+L) (Alexa488)	1:500	Life Technoloies	A21202
Goat anti-mouse IgG2a (Alexa488)	1:500	Life Technoloies	A21131
Goat anti-mouse IgG2b (Alexa488)	1:500	Life Technoloies	A21141
Goat anti-mouse IgG1 (Cy3)	1:500	Jackson ImmunoResearch	115-165-205
Goat anti-mouse IgG (H+L) (Alexa647)	1:500	Life Technoloies	A21236
Goat anti-mouse IgG1 (Alexa647)	1:500	Life Technoloies	A21240
Rat anti-mouse IgG1 (microbeads)	1:5	Miltenyi Biotec	130-047-101
Goat anti-rabbit IgG (H+L) (Alexa488)	1:500	Life Technoloies	A11008
Donkey anti-rabbit IgG (H+L) (Alexa488)	1:500	Life Technoloies	A21206
Donkey anti-rabbit IgG (H+L) (Cy3)	1:500	Jackson ImmunoResearch	711-165-152
Donkey anti-rabbit IgG (H+L) (Alexa647)	1:500	Jackson ImmunoResearch	711-605-152
Donkey anti-rat IgG (H+L) (Alexa488)	1:500	Life Technoloies	A21208

SUPPLEMENTAL EXPERIMENTAL PROCEDURES

Culture of hPSCs

H9 hESCs and 201B7, 585A1 and 604A1 hiPSCs were generally cultured and maintained on mitomycin C-treated STO feeder cells in Primate ES medium (ReproCELL) as described previously (Gotoh et al., 2014).

For the 201B7 and 585A1 hiPSC lines, the cells were alternatively maintained on feeder-free Geltrex (Life Technologies)-coated plates in Essential 8 medium (Life Technologies) supplemented with 50 U/ml of penicillin/streptomycin (Life Technologies) prior to the differentiation studies within 10 passages. The cells cultured in the feeder-free system were passaged with a split ratio of 1:3 or 1:4 after being washed with PBS, incubated in 0.5mM EDTA/PBS for 5 minutes at 37 °C and suspended in pre-warmed Essential 8 medium.

2D differentiation of hPSCs into ventralized anterior foregut endoderm cells (VAFECs)

For the differentiation of hPSCs maintained on mitomycin C-treated STO feeder cells, 70% confluent hPSCs were dissociated and seeded as described previously (Gotoh et

al., 2014).

For the differentiation of feeder free hiPSCs, 80% confluent hiPSCs were incubated in 10 μM of Y-27632 (LC Laboratories) for 1 hour prior to dissociation. The cells were subsequently rinsed carefully with PBS and incubated in Accutase (Innovative Cell Technologies) for 20 minutes at 37°C. The detached hPSCs were then dissociated into single cells via pipetting, and seeded on Geltrex-coated plates at a density of 1.375 $x10^5$ $cells/cm^2$ in Step 1 medium containing RPMI1640 medium (Nacalai Tesque), 1x B27 supplement (Life Technologies, #17504-044), 50 U/ml of penicillin/streptomycin, 100 ng/ml of human activin A (R&D systems) and 1 μM of CHIR99021 (Axon Medchem), supplemented with 10 μM of Y-27632 (day 0-1) and 0.25 mM (day 1) and 0.125 mM (day 2-6) of sodium butyrate (Kajiwara et al., 2012). From Step 2 to Step 3, the basal medium consisted of DMEM/F12 plus GlutaMAX (Life Technologies), 1x B27 supplement, 50 U/ml of penicillin/streptomycin, 0.05 mg/ml of L-ascorbic acid (Wako) and 0.4 mM of monothioglycerol (Wako), as minimally modified from our previous report (Gotoh et al., 2014). On day 6, the medium was changed to Step 2 medium, containing the basal medium with 100 ng/ml of human recombinant Noggin (HumanZyme) and 10 μM of SB-431542 (Stem RD) (Green et al., 2011). On day 10, the medium was changed to Step 3 medium,

containing the basal medium with 20 ng/ml of human recombinant BMP4 (HumanZyme) and the combination of optimal doses of all-trans retinoic acid (ATRA) (Sigma-Aldrich) and CHIR99021; 0.5 μM and 3.5 μM for H9 hESCs, 0.05 μM/2.5 μM for 201B7 hiPSCs, 0.5 μM/3.5 μM for 585A1 hiPSCs and 1.0 μM/2.5 μM for 604A1 hiPSCs.

Isolation of CPM^+ and CPM^- cells from VAFECs

The induced VAFECs were dissociated with Accutase and resuspended in DMEM/F12 containing 2% FBS. The cells were filtered through a 40 μm cell strainer (BD Falcon) to remove cell clumps.

The dissociated cells were washed and resuspended in 1% BSA/PBS, followed by staining with mouse anti-human CPM antibodies (Leica Microsystems) at 4°C for 15 minutes. After rinsing twice with 0.5% BSA/PBS containing 2 mM EDTA, the cells were stained at 4°C for 15 minutes with anti-mouse IgG1 microbeads (Miltenyi Biotec) for CPM^+ cell-sorting or with Alexa 647-conjugated anti-mouse IgG antibody (Life Technologies) for CPM^- cell-sorting. We avoided to sort CPM^- cells by magnetic activated cell sorting (MACS), because a portion of the CPM^+ cells were involved in the CPM^- cell fraction as reported previously (Gotoh et al., 2014).

After washing, the cells were separated using a magnetic stainless column (Miltenyi Biotec) twice or FACSAria II flowcytometer (BD Biosciences). A total of 10 μM of Y-27632 was maintained for all processes until the CPM^+ cells were sorted.

Differentiation of CPM^+ VAFECs into proximal airway epithelial cell (PAEC) spheroids

A total of $4.0x10^5$ cells/cm^2 of CPM^+ cells isolated from the induced VAFECs on day 14 were resuspended in 112 μl of Step 4 medium supplemented with 10 μM of Y-27632 and mixed with an equal volume of growth factor-reduced Matrigel (Coning). In 3D differentiation protocols (Figures 3 and S3, protocols a ~ e), a total volume of 224 μl was carefully pipetted into a 12-well cell culture insert (Corning, #353180) and 1 ml of Step 4 medium was added to the lower chamber and changed every other day for 14 days. On day 28, the medium of the lower chamber was changed to Step 5 medium and replaced every other day until day 42 or day 56.

Step 4 medium consisted of the same basal medium as Steps 2 and 3, and contained 3.0 μM of CHIR99021, 100 ng/ml of FGF10, and 10 μM of Y-27632.

Step 5 medium consisted of PneumaCult-ALI Maintenance medium (STEMCELL

Technologies) supplemented with 10 μM of Y-27632. PneumaCult-ALI Maintenance medium was prepared by adding the attached supplement solution, 4 μg/ml of heparin (Nacalai Tesque) and 1 μM of hydrocortisone (Sigma-Aldrich) to the ready-made basal medium, according to the manufacturer's instructions. 10 μM of Y-27632 was kept to avoid dissociation-induced cell death throughout the induction process from day 14 to the end (Watanabe et al., 2007). For comparison, two ALI protocols (Figure S3A, protocols i and j) were modified from previous reports (Wong et al., 2012; Firth et al., 2012). In one ALI protocol (Figure S3A, protocol i), the medium included FGF18 from day 19 to day 28 and the cells were cultured in ALI condition (Wong et al., 2012). In the other ALI protocol (Figure S3A, protocol j), the cells were cultured in medium containing DAPT as an inhibitor of Notch signaling pathway from day 22 to day 42 in ALI conditions (Firth et al., 2012).

Measurement of the ciliary beat frequency and mucociliary transport

To analyze ciliary beat frequency (CBF) and mucociliary transport, hiPSC-derived multi-ciliated airway cells (MCACs) which were differentiated in 3D Matrigel blocks were dissociated on day 42 and replated on a Geltrex-coated 12-well cell culture insert in a total

volume of 0.5 ml at a density of 7.5 $x10^5$ cells/cm^2 and 1 ml of Step 5 medium plus 10 μM of DAPT was added to the lower chamber and replaced every other day for 14 days before analyses. By reducing the medium to 0.125 ml in the upper chamber, ALI condition was started on day 43. Each sample was placed on the surface of a slide glass and dipped in 100 μl of Step 5 medium.

In the 3D-ALI protocol, the CBF of MCACs in a sheet was examined using an upright microscope (Zeiss Axioplan; Carl Zeiss) with an x63 water immersion objective. In the 3D protocol, the CBF of MCACs in the spheroids was examined by mincing the 3D Matrigel blocks into small pieces on day 42, putting cover slips on the samples and observing ciliary movement using a Zeiss Axioplan microscope with a x63 oil immersion objective. Normal human bronchial epithelial cell (NHBEC) (Lonza) -derived MCACs that were induced in ALI culture or 3D Matrigel blocks in Step 5 medium plus 10 μM of DAPT for 14-28 days were used as positive controls.

For measuring the CBF, 2,048 frames of bright-field images of the beating cilia were acquired at 250 frames per second (fps) on a high-speed video camera (FASTCAM MC2.1; Photron) with the Photron FASTCAM Viewer Ver. 3.0 software program. Then the CBF was calculated using a fast Fourier transform (FFT) based analysis. First, each frame

was subdivided into 1,024 small regions of interests (ROIs) (2.5 x 2.5 μm square) in using the Image J software program (National Institutes of Health). Then, the average brightness was calculated for each ROI and frame, followed by FFT using Scilab software programs (Scilab Enterprises). To minimize the effect of noise, we selected ROIs that included beating cilia by thresholding the CBF within the range of 3 and 35 Hz. A representative image of the selected ROIs is shown in Figure S4F.

To analyze mucociliary transport, the flow of fluorescent beads (a 1000-fold dilution of Fluoresbrite, 0.5 μm; Polysciences) that reflected mucociliary transport was recorded at 9 fps and a total of 128 frames per take were acquired by an Orca-ER CCD camera (Hamamatsu) connected to an upright fluorescent microscope (BX51; Olympus) with x20 objective. Fluorescent beads placed on the ALI-induced MCACs were traced with MTrackJ (Image J plug-in). After confirming the estimated flow velocity of the beads to be approximately 7.4-10.1 μm/s in both hiPSC- and NHBEC-derived MCACs, we evaluated the degree of diffusion of the beads to avoid an inaccurate conclusion affected by velocity generated by fluctuating movements of the beads without orientation. Then, the mean square displacement (MSD) was calculated from the trajectories in each samples and applied to the Einstein-Smoluchowski equation;

$$< r^2 (t) > = 2dDt$$

$< r^2 (t) >$ was regarded as the MSD. Dimension (d) was set as 2, because each trajectory was obtained by two-dimensional image acquisition in the present study. Time (t) was set as 14.2 sec required to take 128 frames at 9 fps. The resulting diffusion coefficient (D) was defined as the mucociliary transport index in the present study. Experiments were performed using hiPSC (201B7, 585A1 and 604A1 hiPSCs)- and NHBEC-derived MCACs for three times independently per cell line. Brownian motion was set as a negative control in analyzing mucociliary transport (Codling et al., 2008). In each experiment, more than 100 cells and 100 trajectories in each hiPSC line, were analyzed for calculating the CBF and mucociliary transport index, respectively. The samples were carried using a portable CO_2 incubator (Corefront) and observed immediately after taking out of the incubators to keep a condition of 5% CO_2 at 37°C.

Quantitative RT-PCR

Total RNA was isolated using the PureLink RNA Mini Kit (Life Technologies). cDNA was synthesized from 80 ng of total RNA using the SuperScript III First-Strand Synthesis System (Life Technologies) and amplified using Power SYBR PCR Master Mix

with the ABI7300 Real-Time PCR System (Life Technologies), as described previously (Gotoh et al., 2014). The PCR reactions were performed in duplicate for each sample. The expression level of each gene was calibrated to that of *β-ACTIN* and compared to the expression level of each gene in the fetal human lung samples (17, 18, and 22 weeks of gestation, Agilent Technologies, #540177, Lot.0006055802), or fetal trachea samples (29 weeks of gestation, Agilent Technologies, #R1244160-10, Lot.B402231). All primer sets are shown in Table S1.

CIF imaging

The fetal human tissue cryosections and cells cultured under 2D condition were fixed with 4% paraformaldehyde /PBS (Nacalai Tesque) for 15 minutes at RT. For immunostaining of Matrigel-embedded 3D spheroids, the samples were fixed with 4% paraformaldehyde for 15 minutes at RT, then after washing with PBS three times, left in 30% sucrose overnight at 4°C. The samples were subsequently embedded in the OCT compound (Sakura Finetek) and sectioned at 10 μm slices. After washing three times with PBS, the samples were permeabilized in 0.2% Triton X-100 /PBS for 15 minutes at RT and blocked with 5% normal donkey serum (Millipore)/1% BSA/PBS for 30 minutes at RT.

Finally, the cells were immunostained with the primary antibodies for 30 minutes at RT and with the secondary antibodies for 30 minutes at RT, as described previously (Gotoh et al., 2014). All primary and secondary antibodies are listed in Table S2. The nuclei were counterstained with Hoechst-33342. All immunofluorescence images were acquired using a TCS SP8 confocal microscope (Leica Microsystems) in order to analyze co-localization of cell lineage markers precisely. The quantification of efficiency was performed by scoring the number of $FOXJ1^+$ cells, $CHGA^+$ cells or SYP^+ cells relative to the total number of nuclei using the MetaMorph 7.7 image analysis software program (Molecular Devices) in an average of five randomly selected fields at 20x magnification (n = 3 independent experiments).

Electron microscopy

Matrigel-embedded epithelial spheroids were incubated in fixative containing 2.5% glutaraldehyde, 4% paraformaldehyde and 0.1M phosphate buffer (pH 7.4) at RT for two hours. For transmission electron microscopy, because the Matrigel-embedded spheroids were slurry and did not easily form pellets, they were re-embedded in the agarose gel. After being washed three times in 0.1 M phosphate buffer (pH 7.4), the samples were

incubated in 1 % osmium tetroxide (Nacalai Tesque) for two hours. Then, the samples were washed in ascending concentrations of ethanol and embedded in epoxy-resin and DMP-30 (Nacalai Tesque). Thin sections were doubly stained with uranyl acetate and lead citrate and examined under a Hitachi H-7650 transmission electron microscope. For scanning electron microscopy, the samples were post fixed in 1 % osmium tetroxide (Nacalai Tesque) for two hours. The samples were then dehydrated, dried, and coated with a thin layer of platinum palladium. Finally, the specimens were examined with a Hitachi S-4700 scanning electron microscope.

Human fetal tissues and total RNA samples

The following human specimens were obtained from DV Biologics (Canada), Agilent Technologies (United States), and BioChain (United States): total RNA of the fetal lungs (17, 18 and 22 weeks of gestation, Agilent Technologies, #540177, Lot.0006055802), another sample of the fetal lung for confirming the gene expression of PNEC markers (36 weeks of gestation, BioChain, #R1244152-50, Lot.B210105), adult lung (40 years of age, Agilent Technologies, #540019, Lot.0006118369), fetal trachea (29 weeks of gestation, Agilent Technologies, #R1244160-10, Lot.B402231), adult trachea (29 years of age,

BioChain, # R1234160-50, Lot.B803066), fetal liver (20 weeks of gestation, BioChain, # R1244149-50, Lot.A601605), and postnatal thymus (6, 6, and 10 months of age, Life Technologies, #AM6000, Lot.1102082), and frozen tissue of the fetal lung (18.5 weeks of gestation, DV Biologics, PP001-FS, Lot.102508RH), fetal liver (28 weeks of gestation, BioChain, T1244149, Lot.B511025) and adult thyroid (25 years of age, BioChain, #T1234265-RT1, Lot.A709031).

SUPPLEMENTAL REFERENCES

Codling EA, Plank MJ, Benhamou S. (2008). Random walk models in biology. J R Soc Interface. *5*, 813-834.

Green MD, Chen A, Nostro MC, d'Souza SL, Schaniel C, Lemischka IR, Gouon-Evans V, Keller G, Snoeck HW. (2011). Generation of anterior foregut endoderm from human embryonic and induced pluripotent stem cells. Nat. Biotechnol. *29*, 267–272.

Kajiwara M, Aoi T, Okita K, Takahashi R, Inoue H, Takayama N, Endo H, Eto K, Toguchida J, Uemoto S, Yamanaka S. (2012). Donor-dependent variations in hepatic differentiation from human-induced pluripotent stem cells. Proc. Natl. Acad. Sci. USA.*109,* 12538-12543.

Watanabe K, Ueno M, Kamiya D, Nishiyama A, Matsumura M, Wataya T, Takahashi JB, Nishikawa S, Nishikawa S, Muguruma K, Sasai Y. (2007). A ROCK inhibitor permits survival of dissociated human embryonic stem cells. Nat. Biotechnol. *25*, 681-686.
